# Heat-response patterns of the heat shock transcription factor family in advanced development stages of wheat (*Triticum aestivum* L.) and thermotolerance-regulation by *TaHsfA2–10*

**DOI:** 10.1186/s12870-020-02555-5

**Published:** 2020-08-03

**Authors:** Xiu-lin Guo, Sai-nan Yuan, Hua-ning Zhang, Yuan-yuan Zhang, Yu-jie Zhang, Gui-yan Wang, Ya-qing Li, Guo-liang Li

**Affiliations:** 1 Institute of Genetics and Physiology, Hebei Academy of Agriculture and Forestry Sciences / Plant Genetic Engineering Center of Hebei Province, No. 598, Heping West Street, Shijiazhuang, 050051 PR China; 2 College of Life Sciences, Hebei Normal University, Shijiazhuang, 050024 PR China; 3 Faculty of Agronomy, Hebei Agricultural University, No. 2596, Lekai South Street, Baoding, 071001 PR China; 4 Shijiazhuang Academy of Agriculture and Forestry Science, No. 479, Shengli North Street, Shijiazhuang, 050000 PR China

**Keywords:** Heat shock transcription factor, Wheat, Eexpression pattern, Thermotolerance, Transcription activity, Binding activity

## Abstract

**Background:**

Heat shock transcription factors (*Hsf*s) are present in majority of plants and play central roles in thermotolerance, transgenerational thermomemory, and many other stress responses. Our previous paper identified at least 82 *H*sf members in a genome-wide study on wheat (*Triticum aestivum* L.). In this study, we analyzed the *Hsf* expression profiles in the advanced development stages of wheat, isolated the markedly heat-responsive gene *TaHsfA2–10* (GenBank accession number MK922287), and characterized this gene and its role in thermotolerance regulation in seedlings of *Arabidopsis thaliana* (L. Heynh.)*.*

**Results:**

In the advanced development stages, wheat *Hsf* family transcription profiles exhibit different expression patterns and varying heat-responses in leaves and roots, and *Hsf*s are constitutively expressed to different degrees under the normal growth conditions. Overall, the majority of group A and B *Hsf*s are expressed in leaves while group C *Hsf*s are expressed at higher levels in roots. The expression of a few *Hsf* genes could not be detected. Heat shock (HS) caused upregulation about a quarter of genes in leaves and roots, while a number of genes were downregulated in response to HS. The highly heat-responsive gene *TaHsfA2–10* was isolated through homeologous cloning. qRT-PCR revealed that *TaHsfA2–10* is expressed in a wide range of tissues and organs of different development stages of wheat under the normal growth conditions. Compared to non-stress treatment, *TaHsfA2–10* was highly upregulated in response to HS, H_2_O_2,_ and salicylic acid (SA), and was downregulated by abscisic acid (ABA) treatment in two-leaf-old seedlings. Transient transfection of tobacco epidermal cells revealed subcellular localization of *TaHsfA2–10* in the nucleus under the normal growth conditions. Phenotypic observation indicated that *TaHsfA2–10* could improve both basal thermotolerance and acquired thermotolerance of transgenic *Arabidopsis thaliana* seedlings and rescue the thermotolerance defect of the T-DNA insertion mutant *athsfa2* during HS. Compared to wild type (WT) seedlings, the *TaHsfA2–10*-overexpressing lines displayed both higher chlorophyll contents and higher survival rates. Yeast one-hybrid assay results revealed that *TaHsfA2–10* had transactivation activity. The expression levels of thermotolerance-related *AtHsps* in the *TaHsfA2–10* transgeinc *Arabidopsis thaliana* were higher than those in WT after HS.

**Conclusions:**

Wheat *Hsf* family members exhibit diversification and specificity of transcription expression patterns in advanced development stages under the normal conditions and after HS. As a markedly responsive transcriptional factor to HS, SA and H_2_O_2_, TaHsfA2–10 involves in thermotolerance regulation of plants through binding to the HS responsive element in promoter domain of relative *Hsps* and upregulating the expression of *Hsp* genes.

## Background

Owing to greenhouse gas emissions, the global mean surface temperature has increased about 0.65 °C from 1956 to 2005 [1]. The rising temperature has become one of the major climatic disasters restricting crop growth and development around the world [2]. Wheat (*Triticum aestivum* L.) is the main cereal crop in many countries of the world and the high and stable yield is the most important breeding target. However, wheat crops frequently suffer from cross-stresses of heat and dry wind, causing recent decreases in both quantity and quality [3]. It is therefore necessary to analyse molecular mechanisms of thermotolerance and develop wheat cultivars with high resistance to heat stress (HS).

Heat shock transcription factors (Hsfs) in plants play central roles in regulating plant thermotolerance. Hsfs can activate the expression of heat shock protein (Hsp) genes and thermotolerance-related genes by binding to HS responsive elements (HSEs) within promoters [4–7]. Since the cloning of yeast *Hsf* in the 1980s, many *Hsfs* have been recently identified at the genome-wide scale in a variety of species [8–12], including the first plant *Hsf* gene from tomato (*Solanum lycopersicum* L.) [13]. Plant Hsfs are divided into group A, B, C and are further divided into several subgroups based on different protein structures [4]. The number of *Hsf* gene family members varies greatly between species. So far, studies have identified 21 *Hsfs* in *Arabidopsis thaliana*, 16 *Hsfs* in tomato, and 82 *Hsfs* in wheat [7, 14].

Most previous studies on *Hsfs* have been limited to A1 and A2 *Hsf* subclasses within the model plants *Arabidopsis thaliana* and *Solanum. Lycopersicum (S. lycopersicum)* [15–18]. The *S. lycopersicum HsfA1* gene is constitutively expressed at low level and the protein coded by the gene localizes to both the nucleus and cytoplasm under the normal growth conditions. HsfA2 is localized in the cytoplasm due to a strong cytoplasmic localization signal, while its nuclear entry relies on the binding of HsfA2 to HsfA1 to form a hetero-oligomer during HS [8, 17]. *HsfA2* expression is strictly induced by HS and HsfA2 proteins can accumulate after continuous or repeated HS and during recovery from HS [8, 17]. Only one *HsfA2* exists in both *Arabidopsis thaliana* and *S. lycopersicum* [13].

*Arabidopsis thaliana* HsfA2 is localized in both the nucleus and the cytoplasm and can activate downstream *Hsp* gene expression upon binding with and activation by AtHsfA1. When *AtHsfA1* is deleted, *AtHsfA2* can enter the nucleus and regulate the expression of a series of *Hsps* and chaperone genes [18]. AtHsfA1 mainly acts as a transcription factor while AtHsfA2 regulates acquired thermotolerance by activating the expression of genes related to reactive oxygen species and carbohydrate and lipid metabolism to maintain cell membrane stability in the later period of HS [19]. In addition, *AtHsfA2* can partially perform certain functions of *AtHsfA1* during exposure to different heat ranges and oxygen stress and can rescue *AtHsfA1* mutant phenotypes [20–22]. Most recently, AtHsfA2 was found to regulate transgenerational thermomemory induced by HS in *Arabidopsis thaliana* by directly activating the H3K27me3 demethylase *REF6* (Relative of early flowing 6) [23], suggesting that HsfA2 may participate in diverse thermotolerance regulation [15, 16, 20, 21, 24].

Studies to determine characteristics and functions of wheat Hsf genes have only recently begun. In 2008, seven *TaHsfs* were identified in wheat, one of which was dramatically upregulated by HS, suggesting that these *TaHsfs* help regulate thermotolerance [25]. In addition, *TaHsfA4a* is upregulated by cadmium stress and participates in cadmium tolerance [26]. Expression of the *TaHsfA2d* gene in *Arabidopsis thaliana* improves thermotolerance, salinity tolerance, and drought tolerance of seedlings, with the seedlings growing at moderately high temperatures displaying increased biomass and yield [27]. For seedlings of *Arabidopsis thaliana* expressing *TaHsf3*, both thermotolerance and cold resistance can potentially be improved [28] In 2014, 56 *Hsf* members from families A, B, and C were identified in *T. aestivum*, many of which are constitutively expressed, and others in subgroups A2, B2, and A6 are significantly upregulated by HS [29]. TaHsfA6f directly regulates the expression of genes *TaHsps*, *TaGAAP* (Golgi anti-apoptotic protein, GAAP), and *TaRof1* (a co-chaperone) and thus enhances seedling thermotolerance [30]. *TaHsfs* vary in expression levels and sensitivity to abiotic stresses including heat, salinity, drought, and cold [31]. *TaHsfC2a* is highly expressed in the filling stage of wheat and its overexpression upregulates the expression of genes related to drought, heat, and abscisic acid (ABA) responses, *TaHsfC2a* also provides proactive heat protection in developing wheat grains via an ABA-mediated regulatory pathway [32].

We previously reported that *TaHsfB2d* can regulate HS responses through a salicylic acid (SA) signalling pathway, which is dependent on H_2_O_2_ levels [33]. Both basal and acquired thermotolerances are improved in *Arabidopsis thaliana* overexpressing *TaHsfA2e*, with increased expression of multiple *Hsp* genes belonging to different *Hsf* group [34]. Hsp genes can improve the thermotolerances of transgenic *Arabidopsis thaliana*, though expression response to HS was different [34]. In another recent report, we identified 82 wheat *Hsf* genes in a genome-wide study. These *TaHsf* family members showed diverse expression patterns in both leaf and roots, and under osmotic stresses such as SA, H_2_O_2_, and ABA in two-leaf-old seedlings of wheat. Among the 82 wheat Hsf genes, 9 members of subclass A2 and 17 members of other subclass were newly identified [14]. However, little is known about the characteristics and functions of these genes nowadays.

The average temperature over land from 2006 to 2015 was 1.53 °C higher than that from 1850 to 1900 and the warming temperature led to reduction of crop yield [35]. It is estimated that the yield of global wheat fall by 6% with 1 °C increasing of global temperature [36]. So it is important to thoroughly investigate *Hsf* gene expression profiles in advanced development period of wheat and understand the thermotolerance-regulating functions of individual *Hsf* members during HS responses. This is especially relevant for subclass A2, which has previously been reported to be important for acquired thermotolerance during advanced development periods of wheat [20]. The aim of this study is to investigate the expression characterization of wheat *Hsf* family in the advanced development stages under HS and further elucidate thermotolerance regulatory function of individual wheat *Hsf*. The results may enable further understanding of biological functions and molecular mechanisms of *Hsf* family members and identify target genes for improving thermotolerance of wheat varieties.

## Results

### Expression patterns of wheat Hsf gene during HS in advanced development stages of *T. aestivum*

**Fig. 1:**
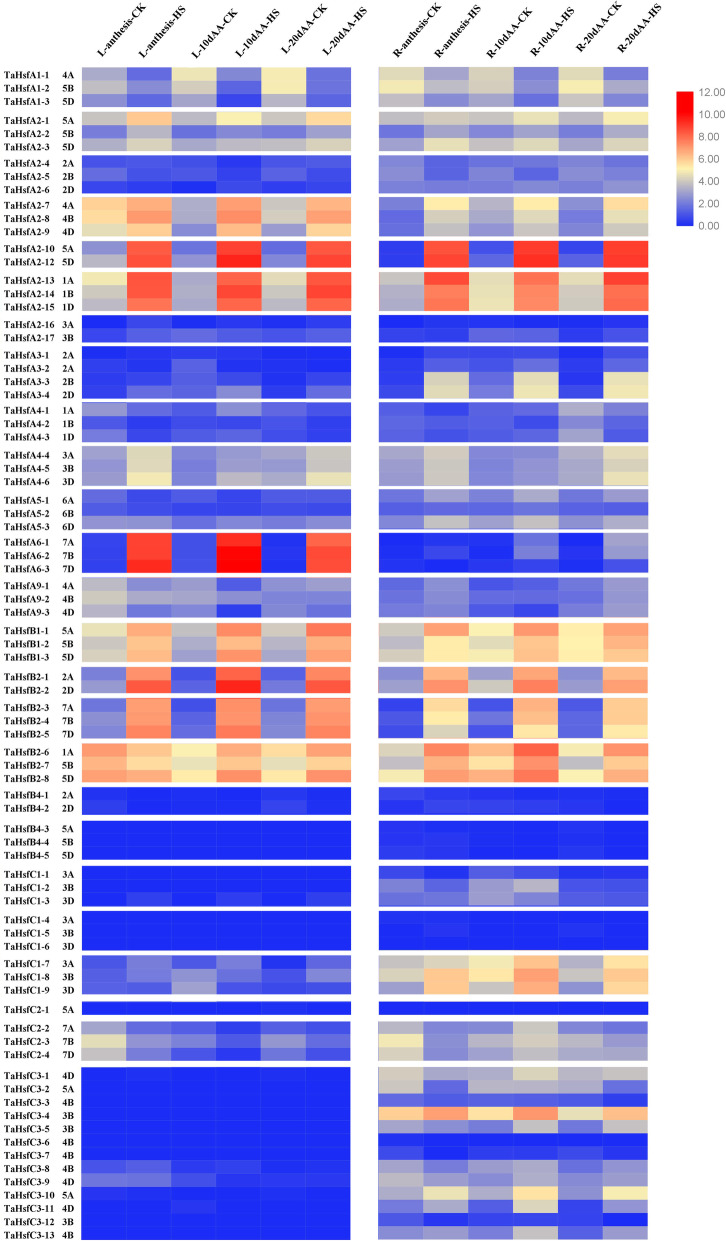
The transcription profiles of genes from wheat *Hsf* family in both leaves (L) and roots (R) of the advanced developmental period under the normal conditions and heat stress (HS). A heatmap was drawn to illustrate the relative expression profiles of 80 *TaHsfs* by TBtools version0.66831. Different colours correspond to log2 transformed values. Red or blue indicates higher or lower relative abundance of each transcript in each sample, respectively. Seedlings of wheat Cang 6005 were grown in the greenhouse with 22 °C/18 °C (day/night), 16 h/8 h photoperiod/dark and 50% humidity for the whole life. The flag leaves and roots were sampled at 60 min and 90 min respectively after heat treatment at anthesis stage (Feekes 10.5.2) and the following 10 days (10d AA), 20 days (20d AA) and used for RNA-Seq analysis. Pooled samples of total 50 individual plants from three pots were collected for each group respectively, and immediately frozen in liquid nitrogen for RNA extraction

Flag leaves and roots of wheat under the normal growth conditions and after HS at 37 °C were sampled at the anthesis stage and latter 10 d and 20 d, and used to analyse expression profiles of wheat *Hsf* genes via RNA-Seq (Fig. 1). Eighty wheat *Hsf* family genes were detected in both leaves and roots, except for *TaHsfA2–11* and *TaHsfA2–18*. Transcription profiles of *TaHsfs* revealed complex expression patterns in leaves and roots. Under the normal conditions, no difference was detected in the expression profiles of most genes in leaves and roots of wheat in different stages. However, some genes were expressed at higher levels in leaves at the anthesis stage than the latter two development stages of wheat. These genes included the subclass A2 members *TaHsfA2–7*, *TaHsfA2–8*, *TaHsfA2–9*, *TaHsfA2–13*, the *TaHsfB1* members, the B2 subclass members of *TaHsfB2–6*, *TaHsfB2–7*, *TaHsfB2–8*, and the C2 subclass members of *TaHsfC2–2*, *TaHsfC2–3*, and *TaHsfC2–4*. Expression levels of *TaHsfA1–1*, *TaHsfA1–2*, and *TaHsfA1–3* increased in leaves in the two development stages after anthesis, and similar expression profiles of *TaHsfB1–1*, *TaHsfB1–2,* and *TaHsfB1–3* were observed in wheat roots. Overall, the majority of class A and B *Hsfs* were expressed at higher levels in leaves while class C *Hsfs* were expressed at higher levels in roots.

*Hsf* expression in *T. aestivum* during advanced development stages exhibited multiple HS response patterns (Fig. 1). In both leaves and roots, *Hsf* expression levels were increased to different degrees under HS, especially those genes of subclasses A2, B1, and B2. Especially, *TaHsfA2–10* and *TaHsfA2–12* were increased most obvious under HS. In contrast, three *TaHsfA1s* were downregulated during HS in leaves and roots of wheat during three development stages. The expression levels of three A6 subclass members were remarkably upregulated by HS in leaves, but not in roots. In addition, the homeologous genes *TaHsfC1–7*, *TaHsfC1–8*, *TaHsfC1–9,* and both *TaHsfC3–4* and *HsfC3–10* were upregulated by HS in roots, but not in leaves. Additionally, the expression of those genes were undetectable during normal and HS conditions, including all subclass B4 members, six subclass C1 members, all subclass C3 members in wheat leaves, all subclass B4 members and three subclass C1 members in roots.

### Amplification of TaHsfA2–10 cDNA and structural analysis of the encoded protein in *T. aestivum*

**Fig. 2:**
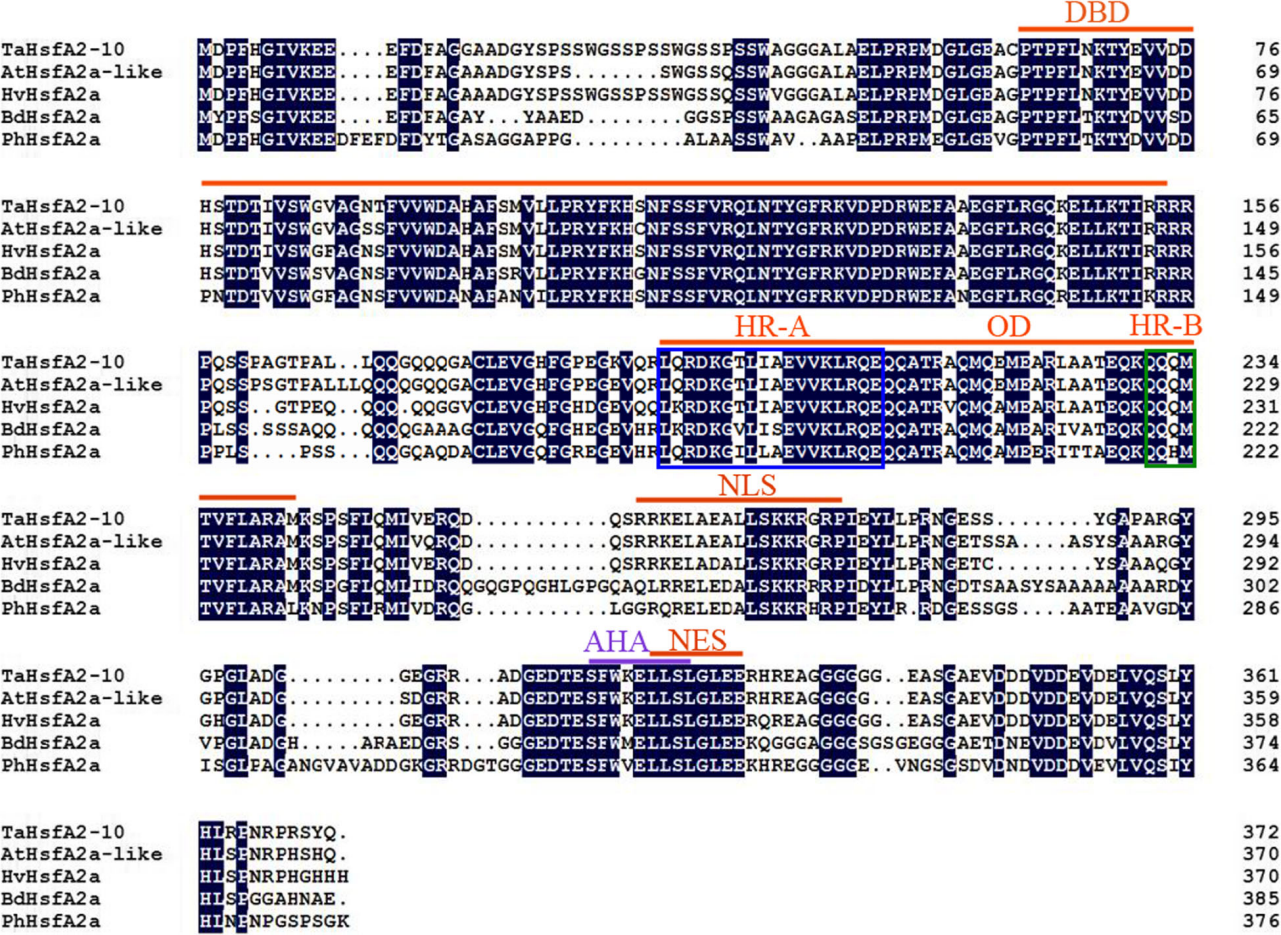
Sequence alignment of TaHsfA2–10 from wheat and HsfA2 proteins in other plant. The TaHsfA2–10 protein sequences were blasted in NCBI (www.ncbi.nlm.nih.gov). The identified protein sequences were aligned with Clustal X 2.0 software, and then the results were minimally repaired by DNAMAN 8.0 (www.lynnon.com) software. TaHsfA2–10: HsfA2–10 from *Triticum aestivum* L., GenBank accession number: QEQ56178; AtHsfA2a-like: HsfA2a-like from *Aegilops tauschii*, GenBank accession number: XP_020200656; HvHsfA2a: HsfA2a from *Hordeum vulgare*, GenBank accession number: BAJ88237; BdHsfA2a: HsfA2a from *Brachypodium distachyon*, GenBank accession number: XP_003559435; PhHsfA2a: HsfA2a from *Panicum hallii*, GenBank accession number: XP_025817582. DBD: DNA-binding domain; HR-A and HR-B: heptad repeats; OD: Oligomerization domain; NLS: nuclear localization signal; NES: nuclear export signal; AHA: activator peptide motif. Black box represents HR-A and green box represents HR-B. Black: same amino acid; White: different amino acid

The cDNA sequence of *TaHsfA2–10* was cloned using homeologous cloning from young leaves of *T. aestivum* Cang 6005 after HS at 37 °C. The full-length sequence of *TaHsfA2–10* is 1119 bp long and encodes 372 amino acids. *TaHsfA2–10*, which is located on chromosome 5AL, is homeologous to previously identified *TaHsfA2–12* on chromosome 5DL [14]. The amino acid sequence of TaHsfA2–10 contained a DNA-binding domain (DBD), an oligomerization domain (OD), a nuclear localization signal (NLS), a nuclear export signal (NES), and an activator peptide motif (AHA). Protein similarity analysis indicated that TaHsfA2*–*10 is highly identical to AtHsfA2a-like from *Aegilops tauschii*, HvHsfA2a from *Hordeum vulgare*, BdHsfA2a from *Brachypodium distachyon*, and PhHsfA2a from *Panicum hallii* (Fig. 2).

### TaHsfA2–10 expression in different tissues and organs of *T. aestivum* under abiotic stress

**Fig. 3:**
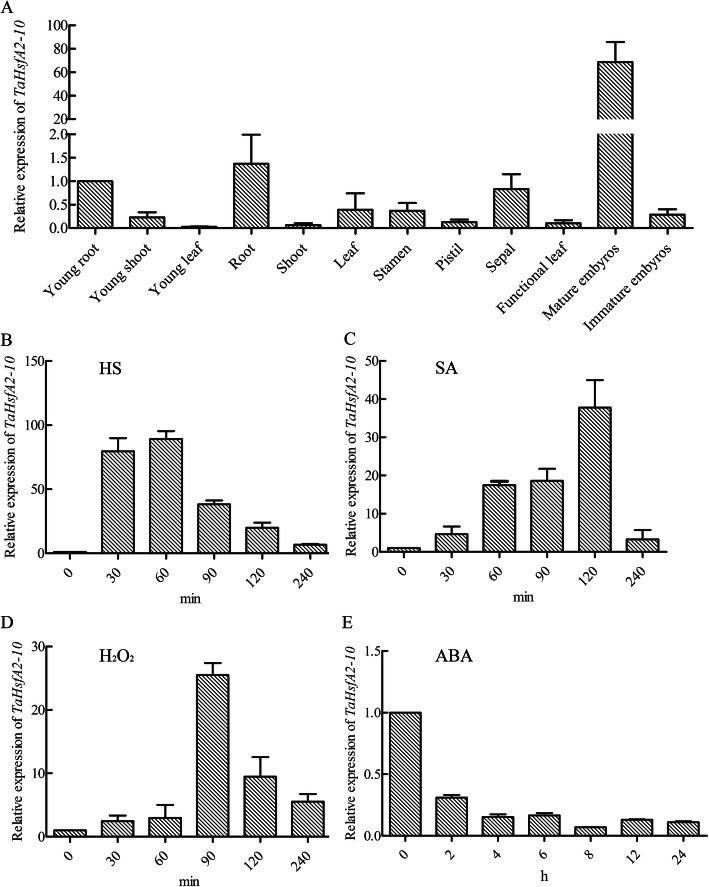
Expression levels of *TaHsfA2–10* in tissues and organs **(a)** and in leaves treated by different time of HS **(b)**, SA **(c)**, H_2_O_2_**(d)** and ABA **(e)**. The two-leaf-old wheat seedlings grown in a growth chamber were subjected to the following treatments: 37 °C HS **(b)**, 0.8 mM SA **(c)**, 10 mM H_2_O_2_**(d)** for 30, 60, 90, 120, 240 min, respectively, and 200 μM ABA **(e)** for 2, 4, 6, 8, 12, 24 h. The new expanding leaves were sampled in different time interval of treatments. Each treatment was repeated three times with totally 40 individual plants sampled each biological experiment, and each biological experiment included three technical replicates. The values of young root and 0 h were normalized as 1 for A and B-E, respectively. The reference gene was *TaRP15.* Each bar value represents mean ± SD of three biological experiments. Raw data refer to Additional file 2

qRT-PCR analysis revealed that *TaHsfA2–10* is constitutively expressed in many tissues and organs in different development stages of *T. aestivum*, with the highest expression levels in mature embryos, and expression levels in other tissues and organs were relative lower, suggesting that *Hsf* genes expression exist tissue-specific variations (Fig. 3a). *TaHsfA2–10* expression levels in leaves were upregulated by HS, peaking at 90 min of the control levels while subjected to HS (Fig. 3b). *TaHsfA2–10* levels also increased after application of exogenous SA (Fig. 3c) and H_2_O_2_ (Fig. 3d) with peak levels nearly 40 times and 25 times of their own controls at 120 min and 90 min after subjected to different stresses, respectively. In contrast, the expression of *TaHsfA2–10* was downregulated by exogenous ABA (Fig. 3e).

### Subcellular localization of TaHsfA2–10

**Fig. 4:**
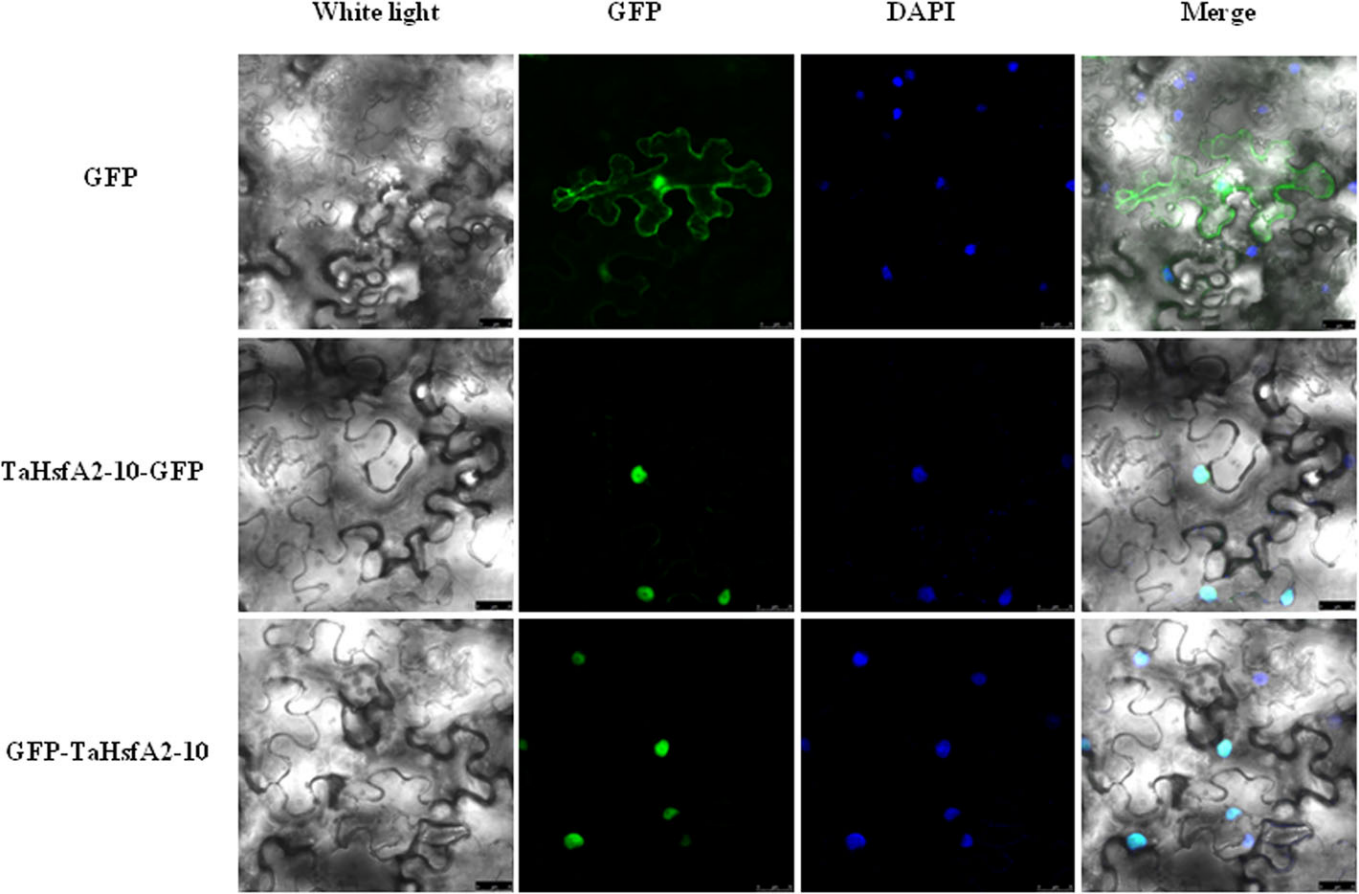
Subcellular localization of TaHsfA2–10 in tobacco epidermal cells under the normal growth conditions. **a** Epidermal cells of tabacco expressing 35S:TaHsfA2–10-hGFP (both C and N terminal fusions) under white light; **b** Epidermal cells of tabacco expressing 35S:TaHsfA2–10-hGFP under green channel florescence (both C and N terminal fusions); **c** Epidermal cells of tabacco expressing 35S:TaHsfA2–10-hGFP under DAPI blue florescence (both C and N terminal fusions); **d** Merge of DAPI and GFP green channel florescence (both C and N terminal fusions)

The recombinant vector of *TaHsfA2–10* with N-terminal of GFP fusion (pCAMBIA1300-TaHsfA2–10-GFP) and the recombinant vector of *TaHsfA2–10* with C-terminal of GFP fusion (pCAMBIA1300-GFP-TaHsfA2–10) were constructed. The two constructs and the empty vector pCAMBIA1300-GFP were infiltrated into tobacco (*Nicotiana tabacum* L.) epidermal cells, respectively. Observation results showed that TaHsfA2–10 was nucleus localized under the normal growth conditions (Fig. 4).

### Analysis of transactivation activity of TaHsfA2–10 in yeast

**Fig. 5:**
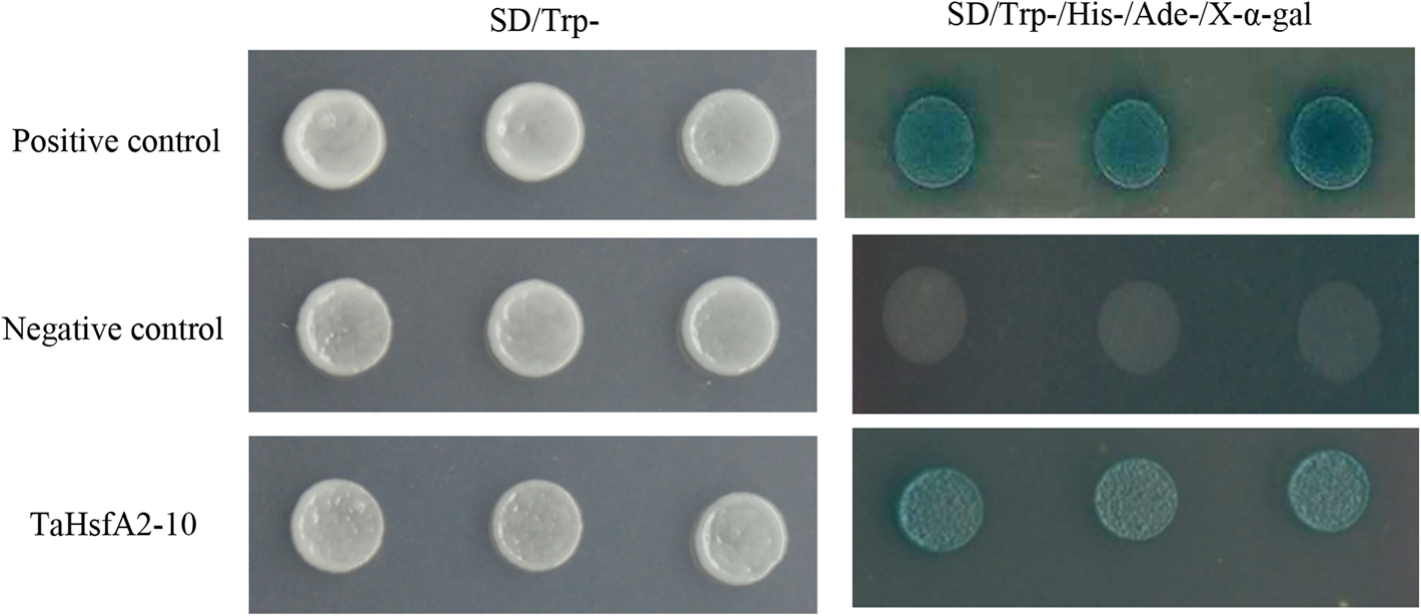
Yeast one-hybrid analysis of *TaHsfA2–10* trans-activation. Positive control, Negative control and *TaHafA2–10* represent yeast cells transformed with pGBKT7–53, pGADT7 and pGBKT7-TaHafA2–10 on the medium of SD/Trp- and SD/Trp−/His−/Ade- (dyed with X-α-gal), respectively

The transactivation activity of TaHsfA2–10 was evaluated in the yeast medium SD/Trp^−^/His^−^/Ade^−^/X-α-gal. As shown in Fig. 5, positive controls containing pGBKT7–53 grew well while the negative control hardly grows. Yeast transformed with pGBKT7-TaHsfA2–10 grew similarly as positive control (Fig. 5). This result suggested that TaHsfA2–10 possesses transactivation activity in yeast.

### Evaluation of thermotolerance regulation by TaHsfA2–10 in transgenic *Arabidopsis thaliana*

**Fig. 6:**
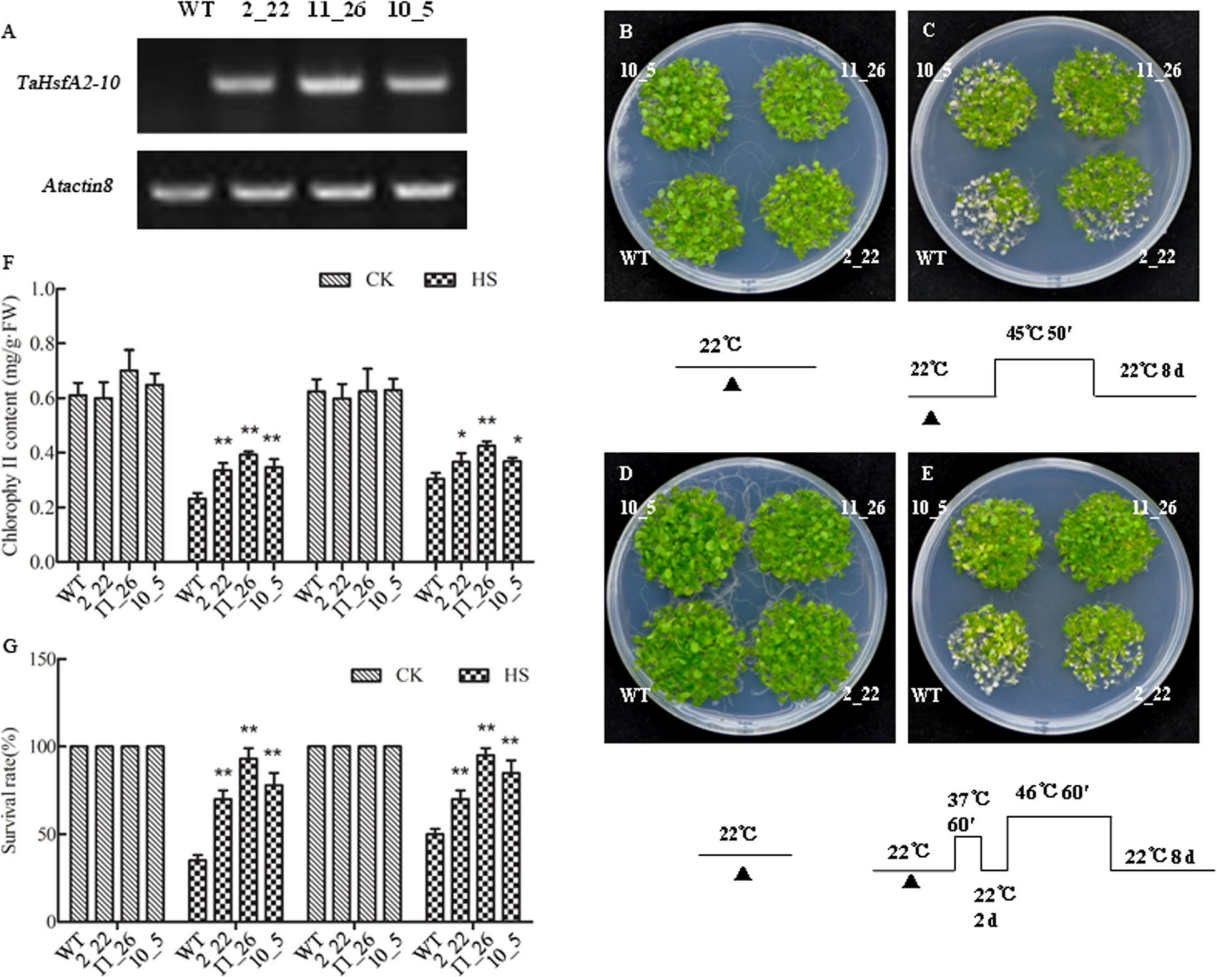
The thermotolerance phenotypes, survival rate and the chlorophyll contents of *TaHsfA2–10* transgenic *Arabidopsis* seedlings and wild type (WT) under the normal conditions and subjected to HS. **a***TaHsfA2–10* relative expression in WT and three transgenic lines of T3 generation by semi-RT-PCR. There were total 50 individual plants of each line of each plate, and the experiment was repeated three times. Single and double asterisks indicate the significant differences between WT and overexpressing lines at *P* < 0.05 and *P* < 0.01 level (*t-test*), respectively. B-E: WT controls and three lines of *TaHsfA2–10* overexpressed *Arabidopsis* (line 2_22, line 10_5 and line 11_26) were used to analyse the basal (BT) and acquired thermotolerances (AT). Five-day-old seedlings (grown in the greenhouse with temperature of 22 °C/18 °C, 16 h light/8 h dark cycles and light of 100 mmol photons m^− 2^ s^− 1^) were treated with different HS regimes listed under each phenotype picture, and the seedlings were recovered at 22 °C for 8 days, then the phenotypes were observed and photographed. **b-c**: assays for BT, **d-e**: assays for the AT. **b, d**: seedlings under the normal conditions; **c, e**: seedlings treated with different HS regimes. After above, the survival rates **(g)** were measured and the rosettes of each line were collected for measurement of chlorophyll contents **(f)**. Total 50 individual plants of each line were divided into three parts and used for chlorophyll contents measurement; three plates were performed for each heat treatment. Each bar value represents mean ± SD of triplicate experiments; raw data refer to Additional file 2 and Additional file 3. Single and double asterisks indicate the significant differences between WT and overexpressing lines at P < 0.05 and P < 0.01 level (*t-test*), respectively

Three transgenic *Arabidopsis* lines overexpressing *TaHsfA2–10* of T3 generation were selected, with semi-RT-PCR confirming *TaHsfA2–10* expression (Fig. 6a). Next, basal and acquired thermotolerance of these *TaHsfA2–10*-expressing *Arabidopsis* seedlings were evaluated with WT seedlings. No obvious phenotypic differences between three transgenic lines and WT plants were observed under the normal growth conditions (Fig. 6b, d); however, the growth vigour of all *TaHsfA2–10*-expressing plants was higher than that of WT controls after two types of HS regimes treatment. Out of the transgenic lines generated, line 11_26 exhibited the strongest basal (Fig. 6c) and acquired thermotolerance phenotypes (Fig. 6e). Chlorophyll levels and survival rates decreased with increasing thermotolerance, but transgenic lines had significantly higher chlorophyll content (Fig. 6f) and survival rates (Fig. 6g) compared to WT under HS conditions. The seedlings of line 11_26 had the highest chlorophyll content (Fig. 6f) and survival rates (Fig. 6g) among the different genotypes.

### Rescued thermotolerance of the *Arabidopsis thaliana* mutant athsfa2 by TaHsfA2–10

**Fig. 7:**
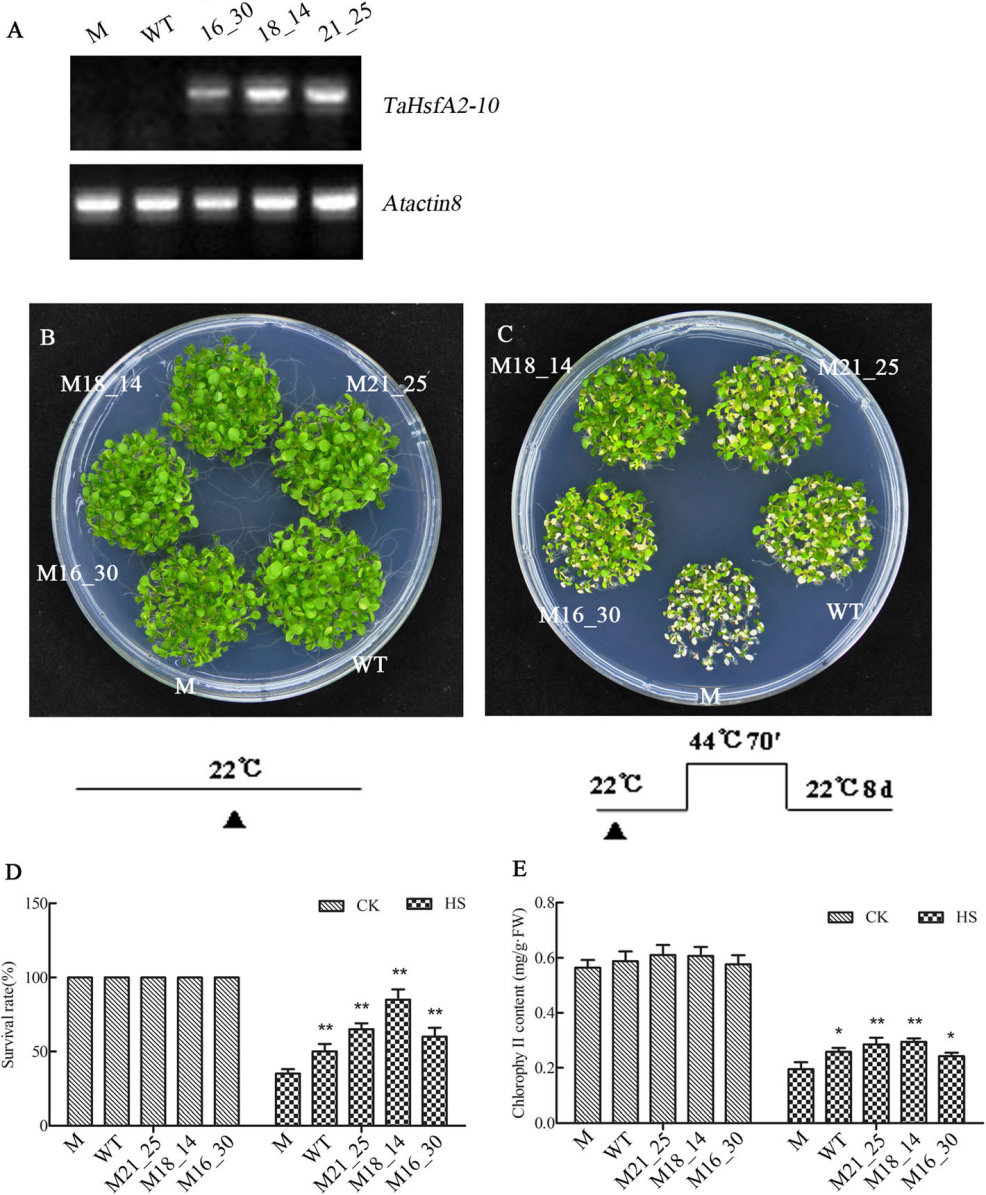
The thermotolerance phenotypes, survival rate and the chlorophyll contents of *atHsfA2–10* recovery *Arabidopsis* seedlings and WT under the normal conditions and HS. **a***TaHsfA2–10* relative expression in mutant (M), WT and three complementary lines of T3 generation by semi-RT PCR; **b-c** WT, *athsfA2* mutant and its three *TaHsfA2–10* complementary homozygous lines (16_30, 18_14 and 21_25) were used to assay the recovery thermotolerances. Five-day-old seedlings were treated with different HS regimes listed under each phenotype picture. After the seedlings were recovered at 22 °C for 8 days, the phenotypes were observed and photographed. After above, the survival rates **(d)** were counted and the rosettes leaves of each line were collected for measurement of chlorophyll contents **(e)**. **b-c** seedlings under the normal conditions and HS; Total 50 individual plants of each line were divided into three parts and used for chlorophyll contents measurement; three plates were performed for each heat treatment. Each bar value represents mean ± SD of triplicate experiments; raw data refer to Additional file 2. Single and double asterisks indicate the significant differences between WT and overexpressing lines at P < 0.05 and P < 0.01 level (*t-test*), respectively

Three *TaHsfA2–10*/*athsfa2* complimentary lines, M16_30, M18_14, M21_25, were created and used to investigate thermotolerance. Semi-RT-PCR analysis confirmed expression of *TaHsfA2–10* in three T3 transgenic lines while WT and the mutant *athsfa2* lacked *TaHsfA2–10* expression (Fig. 7a). Phenotypic observation revealed that growth vigour of WT, *athsfa2*, and *TaHsfA2–10*/*athsfA2* lines were similar under normal growth conditions (Fig. 7b). However, seedlings wilted to different degrees during the recovery period after HS treatment (Fig. 7c). The growth vigour of WT was better than that of the *athsfa2* while complementation lines M16_30 and M21_25 showed similar growth vigour as WT. In addition, the M18_14 line showed the least amount of discolouration, suggesting that *TaHsfA2–10* can rescue the thermotolerance defect of the mutant *athsfa2*. M18_14 also showed higher survival rates and chlorophyll levels compared to WT, *athsfA2* mutant, and M16_30 and M21_25 lines (Fig. 7d, e) after HS treatment.

### TaHsfA2–10-regulating Hsp gene expression is related to HS in *Arabidopsis thaliana*

**Fig. 8:**
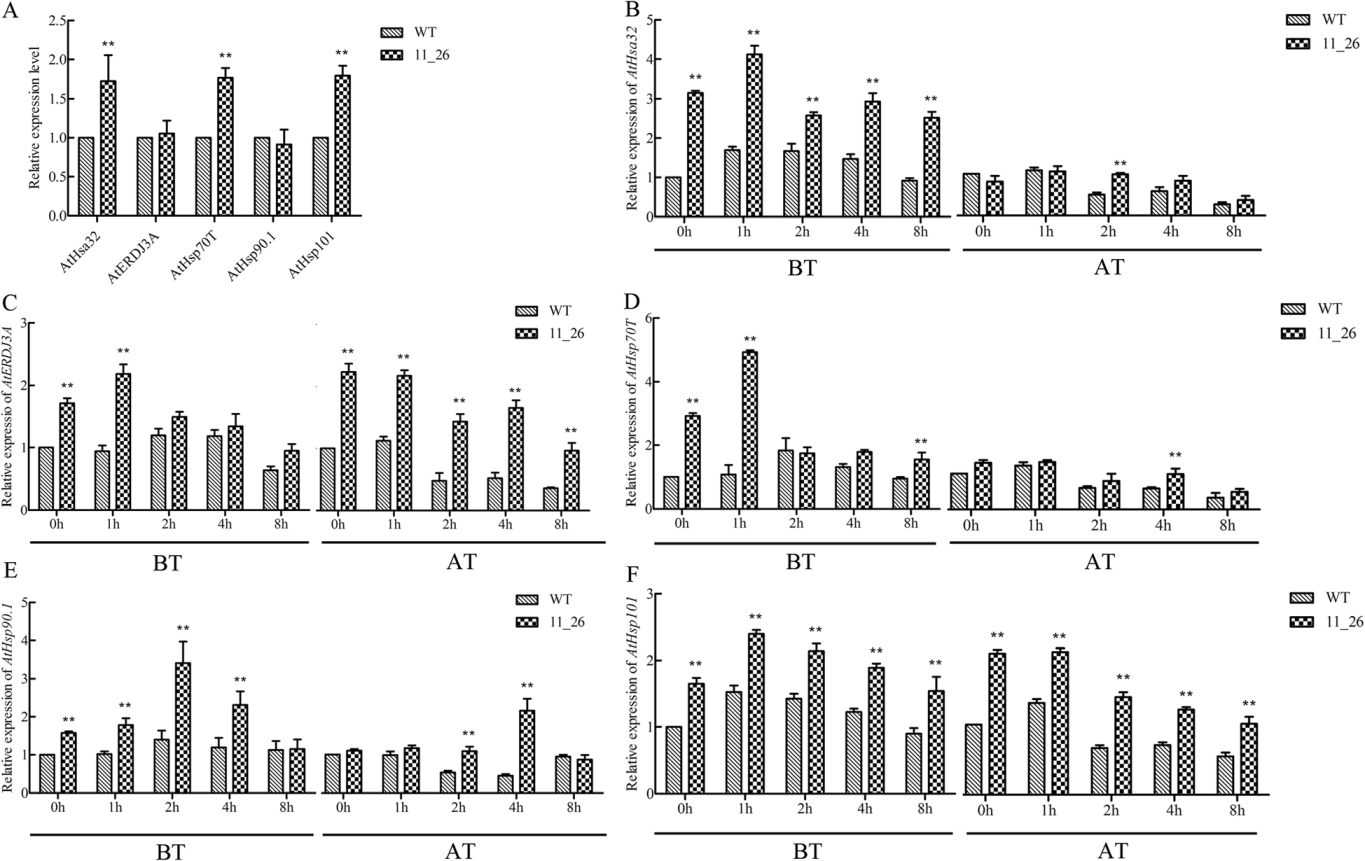
The *Arabidopsis* Hsp gene expression of WT and *TaHsfA2–10-*overexpressed line under normal conditions **(a)** and HS of BT and AT **(b-f)**. Five-day-old T3 generation seedlings of the *TaHsfA2–10* transgenic line 11_26 and WT on agar plates were subjected to HS, and then the rosette leaves were sampled at different time interval for qRT-PCR analysis. Meanwhile, the rosette leaves of the *TaHsfA2–10* transgenic line 11_26 and WT before two kinds of heat treatments were sampled, respectively. For Hsp genes expression of transgenic line under normal conditions, the value of WT was normalized as 1 **(a)**. For the gene expressions of heat treatments **(b-f)**, the value of 0 h was normalized as 1. Each bar value represents mean ± SD of triplicate experiments, three technical replicates were performed in each experiment, and raw data refer to Additional file 2. Double asterisks indicate the significant differences between WT and overexpressing lines at P < 0.01 level (*t-test*)

The expression levels of *Hsps*, including *AtHsa32*, *AtERDJ3A*, *AtHsp70T*, *AtHsp90.1*, and *AtHsp101*, were measured by qRT-PCR. Results showed that the expression levels of these five *AtHsps* in the *TaHsfA2–10* transgenic line 11_26 were slightly higher than that in WT plants under the normal conditions (Fig. 8a). Individual *Hsp* genes were upregulated to different degrees after HS, with peak expression levels appearing 1 h or 2 h after treatment. The expression levels of *AtHsfa32* and *AtHsp70T* were upregulated by 4–5 times during HS in the *TaHsfA2–10* line compared to WT (Fig. 8b-f). After the production of acquired thermotolerance by HS, the expression levels of most *Hsp* genes gradually decreased in both WT and transgenic line 11_26, except for *AtHsp90.1,* which showed higher expression level in line 11_26 than in WT plants 4 h after HS. However, during the recovery periods, the expression levels of *AtHsp90.1* in the transgenic line were higher than those in WT plants. Overall, *Hsp* expression levels were higher after HS that induced basal thermotolerance than HS that induced acquired thermotolerance.

**Fig. 9:**
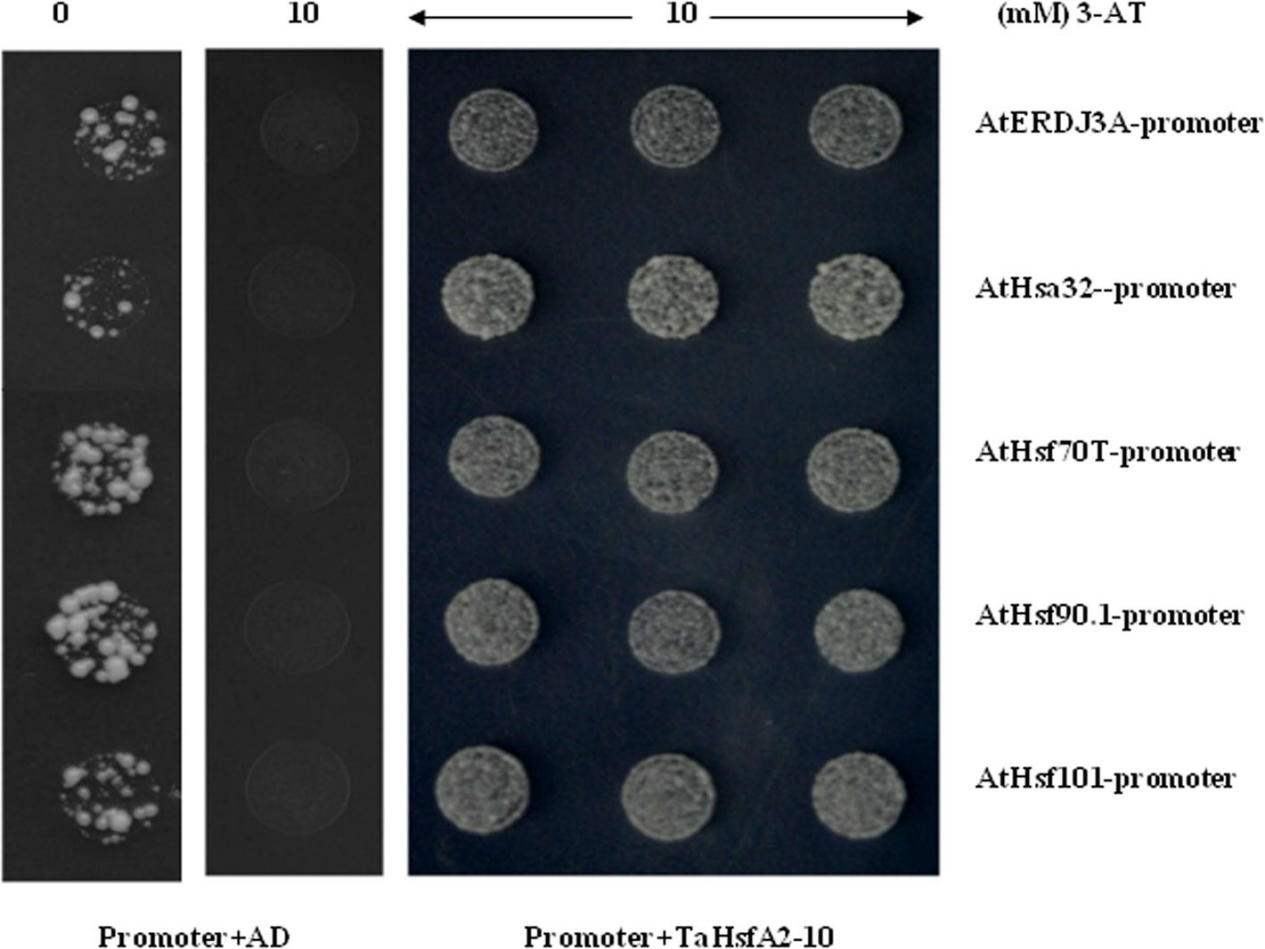
Interaction analysis between *TaHsfA2–10* and the promoters of *AtHsps* in yeast. Promoter +AD: the yeast cells transformed with different pHIS2.1-promoter and the empty vector pGADT7; promoter+TaHsfA2–10: the yeast cells transformed with different pHIS2.1-promoter and the construct pGADT7- *TaHsfA2–10*

Five *AtHsps* were then selected to study the direct binding of HSEs in promoters with TaHsfA2–10 under the normal conditions using the yeast one-hybrid assay. Results revealed that TaHsfA2–10 can bind with HSEs in promoters of all tested *AtHsps* (Fig. 9); further indicating that TaHsfA2–10 can regulate *Hsp* genes expression by binding with their HSEs.

## Discussion

Increasing global temperatures have caused diverse and profound effects on plant growth, development and reproduction [37, 38], and greatly threaten global crop yields. Plants have evolved sophisticated epigenetic machinery to respond quickly to heat [39]. Thermotolerance can be generated upon expression of *Hsp* genes induced by HS. In the advanced development stages of wheat, acquired thermotolerance is the predominant factor determining HS responses [40]. Reports from model plants revealed that members of the subclass *HsfA2s* play central roles in regulating acquired thermotolerance, in recovery from HS, and in transgenerational thermomemory [8, 23]. Therefore, in this study, we identified genes expressed in advanced development stages in *T. aestivum* and evaluated the thermotolerance-regulating roles of individual *Hsf* gene family members.

Our RNA-Seq results reveal that *T. aestivum Hsf* genes exhibit complex expression profiles and heat-response patterns in the advanced stages of wheat development (Fig. 1). The majority of class A and B *Hsf*s were predominantly expressed in wheat leaves while class C *Hsf*s were more highly expressed in wheat roots. Under the normal conditions, no obvious gene expression differences among developmental stages were observed. However, *TaHsfA2–7*, *TaHsfB2–6*, *TaHsfC2–2*, and their two homoeologous genes were more highly expressed during the anthesis stage of leaves. The expression levels of three *TaHsfA1* members increased in leaves of the later developmental stages of wheat, and the same trends were observed for three *TaHsfB1* members in wheat roots. These results indicate that *TaHsf*s are differently expressed among tissue types. The study by Xue et al. [29] revealed that members A2b/c/e, A5b, A6c/d/e were predominantly expressed in the endosperm, subclass B1 members were expressed at higher levels in reproductive organs than in young leaves and young roots, and three C1 and C2 members were highly expressed in embryos of wheat. Most of these genes expression were very low in both leaves and roots of our experiments. However, our results showed that subclass B4 members and three C1 members were nearly undetectable in roots, while Xue’s study indicated that B4 subclass members are expressed in roots and embryos of wheat. We speculate that these differences may be caused by differences in the specific wheat variety examined. Like subclass B4 members, 13 *TaHafC3s* showed very low expression level in leaves but higher in roots in three advanced development stages detected in our experiments under the normal conditions.

RNA-Seq results under HS revealed that the expression of three *TaHsfA1s* was downregulated during HS in both leaves and roots of wheat (Fig. 1), this perhaps caused by sampling time, because the *HsfA1s* always response to heat earlier than *HsfA2s*, and function at early stage of HS [15]. The expression of three *HsfA6s* was upregulated by HS only in wheat leaves at anthesis and two following detective stages, showing tissue-special expression under HS. Wheat *TaHsfA6f* was expressed constitutively in green organs but was markedly up-regulated during HS. *TaHsfA6f* is a transcriptional activator that directly regulates *TaHsps*, *TaGAAP*, and *TaRof1* genes in wheat and its gene regulatory network has a positive impact on thermotolerance [30]. *Arabidopsis* AtHsfA6b operates as a downstream regulator of the ABA-mediated stress response and is required for heat stress resistance, though it response to ABA but not heat [41]. No more reports have been known about *HsfA6s*. Additionally, in our experiment, the expression of the homologues *TaHsfC1–7*, *TaHsfC3–4*, and *TaHsfC3–10* was upregulated only in wheat roots, and the expression levels of subclass B4 members, six members of subclass C1, and 13 subclass C3 members were almost undetectable in leaves during HS while the expression of subclass B4 and three C1 members were almost undetectable in roots in three detected stages of wheat. These results expand those obtained using two-leaf-old wheat seedlings reported by Duan and co-authors, in which the subclass *HsfC3s* mainly responded to ABA [14], suggesting that these genes perhaps mainly participate in ABA signal transduction. These results further support the existence of a proactive *TaHsfC2*-mediated protective mechanism involving an ABA-dependent pathway for regulating heat protection in developing grains of wheat [32]. Our results enrich the expression characterization of wheat *Hsfs* by providing more underlying perceivement on the temporal and spatial expression of wheat *Hsf* family. Results of cis-element analysis showed that majority of *TaHsfCs* promoter contain ABA responsive motifs, only the promoter of *TaHsfC3–1, TaHsfC3–2* and *TaHsfC3–11* contain heat responsive motif (Additional file 1). In addition, *TaHsfB1s* and most *TaHsfB2s* were upregulated in both leaves and roots, suggesting they are involved in heat response of wheat. All *TaHsfB1s* and *TaHsfB2s* contain HSE in their promoter (Additional file 1), revealing these genes can be upstreamly regulated by *Hsfs*. Up to now, few genes are known about *TaHsfBs* function involved in thermotolerance regulation, previous studies showed they serve as coregulators or repressors of the *HsfAs* for lacking a defined activation domain [42]. Zhao et al. reported that *TaHsfB2d* can improve both basal and acquired thermotolerances of transgenic *Arabidopsis thaliana* [33], the *Arabidopsis* seedlings transformed with *CaHsfB2* from *Cicer arietinum* display relatively high drought resistance and thermal tolerance [43]. Lots of work needs to be performed about characteristics and functions of class *HsfBs*.

Studies of model plants indicate that, *HsfA2* members participate in responses to many osmotic stresses, including heat, salt, oxygen, drought, and both ABA- and SA-mediated signal transduction. Once activated by *HsfA1*, *HsfA2* induces the expression of many *Hsp* genes as a key thermotolerance-regulating factor during HS [27]. Among the 82 *Hsf* genes identified in our previous study, most *TaHsfA2s* genes exhibit diverse response patterns to osmotic stresses [14]. In this study, as one of A2 members, *TaHsfA2–10* was shown to be markedly expressed both in leaves and roots under HS at anthesis and later developing stages of wheat (Fig. 1) and in mature embryos (Fig. 3a), also is significantly upregulated by heat, SA, and H_2_O_2_ in two-leaf-old seedlings (Fig. 3b-d), indicating that *TaHsfA2–10* perhaps involve in thermotolerance regulation in wheat different developing stages as a key factor. SA is reported to upregulate *AtHsfA2* expression depending on presence of H_2_O_2_ [44], *TaHsfB2d* regulates HS responses through an SA-mediated signalling pathway in plants which depends on the presence of H_2_O_2_ [33]. *TaHsfC2a* appears to serve a proactive role in heat protection in developing wheat grains via an ABA-mediated regulatory pathway [32]. In both our results and Duan’s report [14], *TaHsfA2–10* expression were downregulated by ABA in two-leaf-old seedlings and later development stages of wheat, speculating that *TaHsfA2–10* perhaps participates in diverse thermotolerance regulation through an SA-mediated signalling pathway but not involving ABA-mediated signal transduction, though the promoter of *TaHsfA2–10* contains both heat and ABA responsive cis-element (Additional file 1). However, whether this pathway dependents on H_2_O_2_ need more researches.

There is only one *HsfA2* gene in both tomato and *Arabidopsis*, tomato HsfA2 was localized in cytoplasm, the nuclear translocation of HsfA2 need to rely on the heterooligomer formed between HsfA2 and HsfA1 [17], while *Arabidopsis* HsfA2 was localized both nuclear and cytoplasm. Different from above, TaHsfA2–10 was confirmed to be localized in nuclear by two constructs of N and C terminal of GFP fusions. We speculate that perhaps nuclear localization enables Hsf to induce downstream genes expression more quickly to improve thermotolerance. Though all contain functional domains such as DBD, NLS, NES, AHA, different localizations of same subclass *Hsf* exist in different species, suggesting diversity and complexity of *Hsf* characteristics and function.

Further phenotype observation provided convincing evidence for the above hypothesis (Figs. 6 and 7). By expressing *TaHsfA2–10* in *Arabidopsis*, we found that *TaHsfA2–10* both improves basal thermotolerance and acquired thermotolerance of the seedlings transgenic *Arbidopsis thaliana*. In addition, *TaHsfA2–10* can rescue the thermotolerance defect of the mutant *athsfa2* during HS. Growing vigour of the *TaHsfA2–10*/*athsfa2* complimentary lines is better than WT, suggesting TaHsfA2–10 perthap has stronger thermotolerance regulation ability than AtHsfA2. The survival rate and chlorophyll contents measurement results provide powerful evidences simultaneously. A previous study demonstrated that thermal tolerance, salinity tolerance, and drought tolerance of *TaHsfA2d*-expressing *Arabidopsis* seedlings were all improved and that seedlings growing at moderately high temperatures could accumulate relatively high amounts of biomass and yield when compared to WT counterparts [27]. Up to now, there is no any report about *TaHsfA2–10*. More diverse gene functions of *TaHsfA2s* need to be deeply investigated in future research.

As molecular chaperones, *Hsp*s play central roles in protecting against stress damage and in assisting with the folding, intracellular distribution, and degradation of proteins [45–47]. *Hsf*s can specifically bind to HSEs in the promoter region of *Hsp* genes as key regulators of *Hsp* genes [4]. Functional HSEs bound by *TaHsfA2b* were previously identified in promoter regions of *TaHsp17*, *TaHsp26.6*, *TaHsp70d*, and *TaHsp90.1-A1*, implying that *TaHsp17* and *TaHsp90.1-A1* are likely direct targets of *TaHsfA2b* [29]. In this study, qRT-PCR of *AtHsp90.1*, *AtHsp70T*, *AtHsp101*, *AtERDJ3A,* and *AtHsa32* showed that these *Hsp* genes were upregulated to different degrees within 4 h of HS, both in WT and transgenic lines (Fig. 8). *AtHsp101* and *AtHsa32* appear involved in long-term acquired thermotolerance in *Arabidopsis* [20, 48, 49], and our results suggest that they also participate in basal thermotolerance. In fact, *TaHsfA2–10* can induce *Hsp* expression in transgenic *Arabidopsis* lines under normal growth conditions, although the resulting expression levels are relatively low (Fig. 8a). In transgenic *Arabidopsis* lines, AtHsfA2 activated the expression of *Hsp* genes like *AtHsp101*, *AtHsfa32*, and *AtHsp-CI*, but not *AtHsp90*, in the absence of *HsfA1* member expression under non-stressed conditions [22]. *TaHsfA2e and TaHsfA2f* dramatically upregulate *AtHsp70T* expression with the improving of basal or acquired thermotolerance [32, 50], and *ZmHsf05* can activate *AtHsp21* and *AtHsp90* expression during HS [24], revealing different Hsfs involves in heat response by activating special *Hsps* expression. Yeast one-hybrid analysis further showed that these detected *Hsp* genes were the direct target genes of TaHsfA2–10 (Fig. 9). These results confirm the regulatory role of TaHsfA2–10 on *Hsp* gene expression during HS and suggest that different *Hsf* members of the same subclass only activate expression of certain *Hsp* genes in different thermotolerance regulation.

## Conclusions

Our results expanded the expression characterization of wheat *Hsf* by acquiring new insights on the underlying mechanisms governing temporal and spatial expression of wheat *Hsf* family members. *TaHsfA2–10* was one of a few markedly responsive genes to HS. TaHsfA2–10 showed transactivation activity in yeast and activated expression of a suite of thermotolerance-related *Hsp* genes in transgenic *Arabidopsis thaliana* plants. *TaHsfA2–10* improved the basal thermotolerance and acquired thermotolerance of transgenic *Arabidopsis* seedlings and rescued the thermotolerance phenotype defect of the mutant *athsfa2* during HS. These findings enrich understanding of the diversity and specificity of *Hsf* expression in wheat. The results may also spur further investigation of the biological functions and molecular mechanisms of *Hsf* family members and the identification of target genes for the genetic improvement of wheat thermotolerance.

## Materials and methods

### Plant materials, growth conditions, and stress treatments

The *T. aestivum* cultivar Cang 6005, used in this study, was provided by the Cangzhou Academy of Agriculture and Forestry Sciences, Hebei province (E116.83, N38.33). This wheat variety is a winter wheat with a total growth period of about 244 days. It has a reputation for heat and salt-tolerance and is mainly planted in the southeast region of Hebei province. Selected seeds were surface sterilized in 0.1% HgCl_2_ for 10 min, rinsed in distilled water repeatedly, and then germinated in a tray. When buds were about 1 cm in size, they were divided into two groups. One group about 30 buds were transplanted into one pot with mesh containing Hoagland nutrient solution, and the other group buds were vernalized at 4 °C for 40 d, then transferred into potted soil (soil:vermiculite, 3:1) in big pots with 8 plants per pot. The plants were cultivated in a greenhouse at 22 °C/18 °C (day/night) with a 16 h/8 h light/dark cycle and 50% humidity under approximately 150 μmol photons m^− 2^ s^− 1^ light intensity. For stress treatments, seedlings with two leaves were treated with HS, H_2_O_2_, SA, or ABA for different time following methods described in Zhao’s paper [33]. For HS treatment, 40 seedlings were put into a new pot containing Hoagland nutrient solution preheated at 37 °C in another chamber, then treated for 30, 60, 90, 120, 240 min. For H_2_O_2_ treatment, 40 seedlings were put into a new pot containing Hoagland nutrient solution with the final concentration of 10 mM H_2_O_2_ for 30, 60, 90, 120, 240 min. For SA treatment, 40 seedlings were put into a new pot containing Hoagland nutrient solution with the final concentration of 10 mM SA for 30, 60, 90, 120, 240 min. For ABA treatment, 40 seedlings were put into a new pot containing Hoagland nutrient solution with the final concentration of 10 mM ABA for 2, 4, 6, 8, 12, 24 h. After stress treatments, the second expanded leaf was obtained from all experiments per treatment. Young root, young shoot and young leaf were sampled at wheat growth stage Feekes 6.0. Root, shoot, leaf, stamen, pistil, sepal and function leaf (flag leaf) were sampled at wheat growth stage Feekes 10.5.2. Immature embryos and mature embryos were obtained at wheat growth stage Feekes 11.1 and Feekes 11.4 respectively. All qRT-PCR results came from three biological experiments and each experiment included three technical replicates. During anthesis (Feekes 10.5.2), 10 daa and 20 daa, pots with 8 plants per pot were transferred to a new growth chamber at 37 °C. Flag leaves and roots of 50 plants were sampled after heat treatment for 60 min (leaves) and 90 min (root) and samples frozen immediately in liquid nitrogen for RNA-Seq analysis of *Hsf* family expression.

The T-DNA insertion mutant line SALK_008978 was provided by Dr. Yee-Yung Charng (Agricultural Biotechnology Research Center, Academia Sinica, Taipei), which was named *athsfa2* derived from the Arabidopsis Biological Resource Center (Ohio State University, USA). Seeds of WT (ecotype Columbia), *athsfa2* and transgenic lines were surface sterilized and sown on MS medium which contained 1% (w/v) sucrose and 0.8% gelrite, then kept at 4 °C for 3 days. Plants were grown to the greenhourse at 22 °C/18 °C (day/night) with a 16 h/8 h light/dark cycle and 50% humidity under approximately 100 μmol photons m^− 2^ s^− 1^ light intensity.

### RNA extraction

Total RNA of different tissues from wheat and *Arabidopsis thaliana* was extracted using the RNarose Reagent Systems kit (Shanghai Huashun Biotechnological Co., Ltd.) according to the manufacturer’s protocol, and genomic DNA contamination was removed by RNase-free DNase I. A NanoDrop 2000 (Thermo Fisher Scientific, Rockford, USA) was used to detect the RNA concentration and quality.

### RNA-Seq analysis of wheat family Hsfs

Flag leaves and roots of anthesis (Feekes 10.5.2) and post-anthesis wheat were sampled for RNA-Seq analysis after stress treatment. RNA-Seq analysis was performed following methods described in [14]. Total RNA of each sample was extracted from 50 plants and genomic DNA was removed by RNase-free DNase I. An Agilent 2100 Bioanalyzer (Agilent Technologies, CA, USA) was used to detect RNA integrity. For RNA sample preparation, about 2 μg RNA of each sample was used as input material. The sequencing libraries were prepared for Illumina by VAHTSTM mRNA-seq V2 Library Prep Kit. The paired-end sequencing of the library was carried out by the HiSeq Xten sequencers (Illumina, San Diego, CA, USA). The sequenced data quality was evaluated by FastQC (version 0.11.2). And the raw reads were selected by Trimmomatic (version 0.36). The clean reads to the wheat reference genome was mapped by HISAT2 (version 2.0) using default parameters. The gene expression abundance of the transcripts was calculated by String Tie (version 1.3.3b). DEGs (differentially expressed genes) were determined by DESeq2 (version 1.12.4). Each sample was detected by RNA-Seq analysis once. A heatmap was drawn to illustrate the relative expression profiles of wheat *TaHsfs* by TBtools version0.66831 [51].

### Cloning of TaHsfA2–10 cDNA and sequence analysis

A total of 1 μg purified RNA was used to synthesize first-strand cDNA using the SuperScript IV First-Strand Synthesis System (Invitrogen). The primers used were: forward primer: 5′-CGGGTTTGGTTCTTTGGA-3′; reverse primer: 5′- CCTTCATCTTCTTTCGCTCA-3′. In addition, the high-fidelity enzyme Pyrobest (TaKaRa) was used for PCR amplification. The PCR system and the reaction procedures were performed according to methods described in [33]. The reaction mixture contained 1× reaction buffer, 2.5 mM dNTP mixture, 1 μL first-strand cDNA, 20 μM forward primer, 20 μM reverse primer and 2 U DNA polymerase in a total volum of 50 μL. The reaction procedure were: 1 min at 94 °C, 32 cycles of 10 s at 98 °C, 30 s at 56 °C, 1 min at 72 °C, and final extension 5 min at 72 °C.

### Expression analysis by quantitative real-time PCR

For the expression analysis of *TaHsfA2–10* in wheat, the specific primers for amplifying *TaHsfA2–10* were designed based on the sequence of 5′-UTR (Forward primer: 5′-CACCTTCGGGTAGCCCCTG-3′, Reverse primer: 5′- GAAAATGTCGCCCTCCTC-3′). The internal reference gene was *TaRP15* (F: 5′-GCACACGTGCTTTGCAGATAAG-3′; R: 5′-GCCCTCAAGCTCAACCATAACT-3′) [29]. The expression level in young roots was set to 1 for the tissue-specific expression analysis and the expression level at 0 h was set as 1 for the stress treatments of wheat. For the expression of *AtHsps* in *Arabidopsis thaliana*, the *TaHsfA2–10* transgenic line 11_26 (T3 generation homozygote) was used. Rosette leaves of the 5-day-old *Arabidopsis* seedlings were sampled at 0 h, 1 h, 2 h, 4 h, and 8 h after heat treatment, as described in the thermotolerance assay section. Five *Arabidopsis* Hsp genes were selected for expression analysis. The internal reference gene was *AtActin8* and the expression level of WT at 0 h was set as 1. Primers used are listed in Additional file 4.

PCR reactions were 20 μL in total: 10 μL SYBR Premix Ex *Taq*II, 0.8 μL 10 μM forward primer, 0.8 μL 10 μM reverse primer, 1 μL 1st strand cDNA, and 7.4 μL ddH_2_O. PCR reactions were performed using a 7500 Real-time PCR System (Applied Biosystems, USA) and reaction procedures carried out according to methods described in [33]. PCR reactions were predenaturated at 95 °C for 30 s, then performed 40 cycles of 5 s at 95 °C and 34 s at 60 °C. The data were analyzed using the 2^-∆∆Ct^ method after the reaction. Each group of experiments included three biological replicates and each biological sample included three technical replicates. The data are represented by mean values ± standard error of three biological replicates for each experiment.

### Determination of TaHsfA2–10 subcellular localization using transient expression in tobacco epidermal cells

For N-terminal fusions of TaHsfA2–10 with GFP, specific primers (Forward primer was 5′-GACGAGCTGTACAAGGAGCTC**ATGGACCCCTTTCAC**-3′ and reverse primer was 5′-CGATCGGGGAAATTCGAGCTC**TCATGGTAGCTGCGGG**-3′. Underlined letters were restriction enzyme sites *Sac*I respectively and bold letters belonged to coding sequence of *TaHsfA2–10*.) were designed to amplify the coding region of TaHsfA2–10 by PCR. The product of PCR was ligated into the vector pCAMBIA1300-GFP digestion with the restriction enzymes *Sac*I (The plasmid map was Additional file 6B). For C-terminal fusions of TaHsfA2–10 with GFP, specific primers (Forward primer was 5′-GAGAACACGGGGGACTCTAGA**ATGGACCCCTTTCAC**-3′ and reverse primer was 5′-GCCCTTGCTCACCATGGATCC**CTGGTAGCTGCGGGGC**-3′. Underlined letters were restriction enzyme sites *Xba*I and *Bam*HI respectively, and bold letters belonged to coding sequence of *TaHsfA2–10*.) were used to amplify the coding sequence of *TaHsfA2–10*, which was then then ligated into the expression vector pCAMBIA1300-GFP after digestion with the restriction enzymes *Xba*I and *Bam*HI (The plasmid map was Additional file 6C). The recombinants driven by 35S CaMV promoter were constructed according to the manufacturer’s protocol using the ClonExpress II kit (Vazyme, Nanjing, China) and transformed into *Agrobacterium tumefaciens* EHA105 cells, which were then used for tobacco epidermal cell infiltration. The empty vector pCAMBIA1300-GFP was as control to study where only the GFP was expressed. Treated tobacco seedlings were grown in a greenhouse with a 16 h/8 h day/night cycle (23 °C/19 °C) under 150 μmol s^− 1^ m^− 2^ light intensity and 50% relative humidity for 3 d. After tobacco epidermal cells were stained with 10 μg/mL DAPI for 5 min and rinsed with physiological saline, the fluorescence of the stained epidermis was examined using the Confocal Zeiss Microsystems META510 (Zeiss, Oberkochen, Germany).

### Transcription activation activity and one-hybrid assays in yeast

Transcription activation activity assays were performed in yeast according to the manufacture’s protocol (TaKaRa, Dalian, China). The coding regions of *TaHsfA2–10* were cloned by PCR using primers (Forward primer was 5′-GAGGAGGACCTGCATATG**ATGGACCCCTTTCAC**-3′ and reverse primer was 5′-GTTATGCGGCCGCTGCAG**TCACTGGTAGCTGCG**-3′. Underlined letters were restriction enzyme sites *Nde*I and *Pst*I respectively, bold letters belonged to coding sequence of *TaHsfA2–10*.) was constructed into the yeast expression vector pGBKT7 digestion with *Nde*I and *Pst*I (The plasmid map was Additional file 6D). The constructs driven by T7 promoter, the pGBKT7–53 as positive control or the empty vector pGBKT7 as negative control with pGADT7 respectively were transformed into AH109, the yeast cell. The yeast cells in exponential growth were diluted to OD_600_ of 0.1 and grown on the deficiency medium plates of SD/Trp^−^/His^−^/Ade^−^/X-α-gal. Then the plates were placed at 30 °C until the yeast cells grew well. Finally, the yeast cells were photographed after 3–5 days.

Yeast one-hybrid assays were performed to detect the binding activity between TaHsfA2–10 and promoters of *AtHsps* according to the methods described by Li et al. [24]. Briefly, the coding region of TaHsfA2–10 was obtained by PCR using primers (Forward primer was 5′-GCCATGGAGGCCAGTGAATTC**ATGGACCCCTTTCAC**-3′ and reverse primer was 5′-CAGCTCGAGCTCGATGGATCC**TCACTGGTAGCTGCG**-3′. Underlined letters were restriction enzyme sites *Eco*RI and *Bam*HI respectively, bold letters belonged to coding sequence of *TaHsfA2–10*.) was contructed into vector pGADT7 digestion with *Eco*RI and *Bam*HI (The plasmid map was Additional file 6E). The promoter sequences of different *AtHsps* were cloned by PCR using primers (Additional file 5) and constructed into vector pHIS2.1 digestion with *Eco*RI and *Sac*I (The plasmid map was Additional file 6F). The pGADT7-TaHsfA2–10 driven by T7 promoter and different constructs of pHIS2.1-promoter driven by minimal HIS3 promoter were transformed into the yeast cell Y187. The SD/Trp^−^/Leu^−^/His^−^ selective medium containing 3-AT (3-amino-1,2,4-triazole) were used in the assay. The yeast cells grew at 30 °C for 3–5 days before they were photographed.

### Generation of transgenic *Arabidopsis thaliana* lines

WT and T-DNA insertion mutant *athsfa2* (SALK_008978, the Arabidopsis Biological Resource Center, Ohio State University) plants of *Arabidopsis thaliana* (ecotype Columbia) were used for genetic transformation. Seeds were surface sterilized with 75% alcohol for 30 s then with 10% sodium hypochlorite for 10 min. Sterile seeds were sown on 0.5x Murashige and Skoog (MS) medium (containing 1% sucrose and 0.8% gelrite, San-EiGenFFI Inc., Osaka, Japan, 1x MS salts and vitamins, pH 5.8) in plastic Petri dishes. After incubation for 3 days at 4 °C in the dark to ensure synchronized germination, plants were grown in a growth chamber under normal conditions (22 °C/18 °C with 16 h light/8 h dark cycles and light intensity at 100 mmol photons m^− 2^ s^− 1^).

The coding region of *TaHsfA2–10* was amplified by PCR using the primers (Forward primer: 5′-GAGAACACGGGGGACTCTAGA**ATGGACCCCTTTCACGGC**-3′, Reverse primer: 5′-CGATCGGGGAAATTCGAGCTCT**CACTGGTAGCTGCGGGG**-3′. Underlined letters were restriction enzyme sites *Xba*I and *Sac*I respectively, bold letters belonged to coding sequence of *TaHsfA2–10*.). The products of PCR were purified and cloned into the binary vector pCAMBIA1300 after digesting the destination plasmid with *Xba*I and *Sac*I (The plasmid map was Additional file 6A). The resulting constructs driven by 35S CaMV promoter were transformed into *Agrobacterium tumefaciens* strain GV3101. Constructs were then transformed into WT and the *Arabidopsis thaliana* mutant *athsfa2* plants using the floral dip method under vacuum conditions as described by Clough et al. [52]. All transgenic plants were selected on MS plates containing 25 mg/mL hygromycin until T3 generation homozygous lines were screened.

### RT–PCR analyses of transgenic lines

Samples of 100 ng of purified mRNA were used for synthesis of the first cDNA strand using Reverse-transcription RT Kit (Invitrogen, Carlsbad, CA, USA). All polymerase chain reactions were performed with Pyrobest DNA Polymerase (Takara Biotech Co. Ltd) in a total volume of 25 mL reaction mixture consisting of 10 × Pyrobest buffer, 2.5 mL; 2.5 mM dNTP mixture, 2 mL; 1st strand cDNA, 2 mL; 20 mM forward primer, 0.25 mL; 20 mM reverse primer, 0.25 mL; Pyrobest DNA polymerase, 0.25 mL; ddH_2_O, 17.75 mL (Forward primer, 5′-ACGCCCTTCCTGAACAAG-3′, Reverse primer, 5′- ATCTGCTGCTGCTTCTGC − 3′). The internal reference gene was *Atactin8* (Forward primer: 5′- CTATTGTCTGTGACAATGG-3′; Reverse primer: 5′- **AACCCTCGTAGATAGGCA** − 3′). The reaction program was as follows: 98 °C for 10 s; 55 °C for 5 s; 72 °C for 2 min, 30 cycles. The products were ligated into the T-vector (pEasy-blunt simple cloning kit, TransGen Biotech, Beijing, China) for sequencing (Shanghai Biotech Co.).

### Thermotolerance assays

For basal thermotolerance assays, WT, mutant *athsfa2*, and three independent T3 generation homozygous transgenic *Arabidopsis* lines were used. For basal thermotolerance, 5-day-old seedlings of WT and *TaHsfA2–10* transgenic lines and on agar plates were subjected to heat shock for 50 min at 45 °C. For acquired thermotolerance assays, 5-day-old seedlings of WT and *TaHsfA2–10* transgenic lines on agar plates were kept at 37 °C for 60 min, then recovered for 2 d at 22 °C and subjected to HS for 60 min at 46 °C. For rescued thermotolerance assays, 5-day-old WT, the mutant *athsfa2*, and *TaHsfA2–10* complementary line seedlings on agar plates were subjected to HS for 70 min at 44 °C, and then allowed to continue growth for 8 days at 22 °C and photographs were taken. More than 50 plants of each line were used per plate and experiments repeated three times.

### Measurements of chlorophyll content

Chlorophyll content was spectrophotometrically measured as previously described by Li et al. [53]. About 0.2 g fresh leaves of *Arabidopsis thaliana* were taken into a capped test tube containing 20 mL acetone and ethanol mixture (acetone:ethanol:ddH_2_O, 4.5:4.5:1.0). The homogenate was filtered after the leaves were completely blenched. The content of Chlorophyll a and Chlorophyll b were calculated according to the value of A645 and A663 of the filtrate respectively.

## Supplementary information

**Additional file 1 **Cis-elements in the promoter of *TaHsf* family.

**Additional file 2.** Raw data of Fig. 3, Fig. 6, Fig. 7 and Fig. 8.

**Additional file 3.** Original, unprocessed versions of the blots in Figs. 6 and 7.

**Additional file 4 **The primers for *Arabidopsis* Hsp genes related to thermotolerance in qRT-PCR.

**Additional file 5 **The primers of the promoters of *AtHsps* used in yeast one hybrid.

**Additional file 6.** The vector maps used in this paper.

## Data Availability

The dataset supporting the conclusions of this article is available in the NCBI-SRA repository, [PRJNA604299 in https://www.ncbi.nlm.nih.gov/bioproject/PRJNA604299], the article and its additional files.

## Abbreviations

Hsf: Heat shock transcription factor
HS: Heat shock
SA: Salicylic acid
ABA: Abscisic acid
WT: Wild type
Hsp: Heat shock protein
HSE: HS responsive element
DBD: DNA-binding domain
OD: Oligomerization domain
NLS: Nuclear localization signal
NES: Nuclear export signal
AHA: Activator peptide motif

## References

[1] Piao S, Ciais P, Huang Y, Shen Z, Peng S, Li J, Zhou L, Liu H, Ma Y, Ding Y (2010). The impacts of climate change on water resources and agriculture in China. Nature..

[2] Solomon S, Qin DH (2007). The physical science basis.

[3] Kang S, Eltahir EAB (2018). North China Plain threatened by deadly heatwaves due to climate change and irrigation. Nat Commun.

[4] Nover L, Scharf KD, Gagliardi D, Vergne P, Czarnecka-Verner E, Gurley WB (1996). The Hsf world: classification and properties of plant heat stress transcription factors. Cell Stress Chaperones.

[5] Nover L, Bharti K, Doring P, Mishra SK, Ganguli A, Scharf KD (2001). Arabidopsis and the heat stress transcription factor world: how many heat stress transcription factors do we need?. Cell Stress Chaperones.

[6] Kotak S, Larkindale J, Lee U, von Koskull-Doring P, Vierling E, Scharf KD (2007). Complexity of the heat stress response in plants. Curr Opin Plant Biol.

[7] Guo M, Liu JH, Ma X, Luo DX, Gong ZH, Lu MH (2016). The plant heat stress transcription factors (HSFs): structure, regulation, and function in response to abiotic stresses. Front Plant Sci.

[8] Scharf KD, Heider H, Hohfeld I, Lyck R, Schmidt E, Nover L (1998). The tomato Hsf system: HsfA2 needs interaction with HsfA1 for efficient nuclear import and may be localized in cytoplasmic heat stress granules. Mol Cell Biochem.

[9] Ikeda M, Ohme-Takagi M (2009). A novel group of transcriptional repressors in Arabidopsis. Plant Cell Physiol.

[10] Mittal D, Chakrabarti S, Sarkar A, Singh A, Grover A (2009). Heat shock factor gene family in rice: genomic organization and transcript expression profiling in response to high temperature, low temperature and oxidative stresses. Plant Physiol Biochem.

[11] Lin YX, Jiang HY, Chu ZX, Tang XL, Zhu SW, Cheng BJ (2011). Genome-wide identification, classification and analysis of heat shock transcription factor family in maize. BMC Genomics.

[12] Tang R, Zhu W, Song X, Lin X, Cai J, Wang M, Yang Q (2016). Genome-wide identification and function analyses of heat shock transcription factors in potato. Front Plant Sci.

[13] Scharf KD, Rose S, Zott W, Schoffl F, Nover L (1990). Three tomato genes code for heat stress transcription factors with a region of remarkable homology to the DNA-binding domain of the yeast HSF. EMBO J.

[14] Duan S, Liu B, Zhang Y, Li G, Guo X (2019). Genome-wide identification and abiotic stress-responsive pattern of heat shock transcription factor family in Triticum aestivum L. BMC Genomics.

[15] Liu HC, Liao HT, Charng YY (2011). The role of class A1 heat shock factors (HSFA1s) in response to heat and other stresses in Arabidopsis. Plant Cell Environ.

[16] Nishizawa A, Yabuta Y, Yoshida E, Maruta T, Yoshimura K, Shigeoka S (2006). Arabidopsis heat shock transcription factor A2 as a key regulator in response to several types of environmental stress. Plant J.

[17] Heerklotz D, Doring P, Bonzelius F, Winkelhaus S, Nover L (2001). The balance of nuclear import and export determines the intracellular distribution and function of tomato heat stress transcription factor HsfA2. Mol Cell Biochem.

[18] Mishra SK, Tripp J, Winkelhaus S, Tschiersch B, Theres K, Nover L, Scharf KD (2002). In the complex family of heat stress transcription factors, HsfA1 has a unique role as master regulator of thermotolerance in tomato. Genes Dev.

[19] Li CG, Chen QJ, Gao XQ, Qi BS, Chen NZ, Xu SM, Chen J, Wang XC (2005). Heat shock transcription factor AtHsfA2 regulating genes expression related to stresses and increase endurance to heat and oxidation stress in Arabidopsis. Sci China Ser C Life Sci.

[20] Charng YY, Liu HC, Liu NY, Chi WT, Wang CN, Chang SH, Wang TT (2007). A heat-inducible transcription factor, HsfA2, is required for extension of acquired thermotolerance in Arabidopsis. Plant Physiol.

[21] Schramm F, Ganguli A, Kiehlmann E, Englich G, Walch D, von Koskull-Doring P (2006). The heat stress transcription factor HsfA2 serves as a regulatory amplifier of a subset of genes in the heat stress response in Arabidopsis. Plant Mol Biol.

[22] Liu HC, Charng YY (2013). Common and distinct functions of Arabidopsis class A1 and A2 heat shock factors in diverse abiotic stress responses and development. Plant Physiol.

[23] Liu J, Feng L, Gu X, Deng X, Qiu Q, Li Q, Zhang Y, Wang M, Deng Y, Wang E (2019). An H3K27me3 demethylase-HSFA2 regulatory loop orchestrates transgenerational thermomemory in Arabidopsis. Cell Res.

[24] Li GL, Zhang HN, Shao H, Wang GY, Zhang YY, Zhang YJ, Zhao LN, Guo XL, Sheteiwy MS (2019). ZmHsf05, a new heat shock transcription factor from Zea mays L. improves thermotolerance in Arabidopsis thaliana and rescues thermotolerance defects of the athsfa2 mutant. Plant Sci.

[25] Qin D, Wu H, Peng H, Yao Y, Ni Z, Li Z, Zhou C, Sun Q (2008). Heat stress-responsive transcriptome analysis in heat susceptible and tolerant wheat (Triticum aestivum L.) by using Wheat Genome Array. BMC Genomics.

[26] Shim D, Hwang JU, Lee J, Lee S, Choi Y, An G, Martinoia E, Lee Y (2009). Orthologs of the class A4 heat shock transcription factor HsfA4a confer cadmium tolerance in wheat and rice. Plant Cell.

[27] Chauhan H, Khurana N, Agarwal P, Khurana JP, Khurana P (2013). A seed preferential heat shock transcription factor from wheat provides abiotic stress tolerance and yield enhancement in transgenic Arabidopsis under heat stress environment. PLoS One.

[28] Zhang SX, Xu ZS, Li PS, Yang L, Wei YQ, Chen M, Li LC, Zhang GS, Ma YZ (2013). Overexpression of TaHSF3 in transgenic Arabidopsis enhances tolerance to extreme temperatures. Plant Mol Biol Report.

[29] Xue GP, Sadat S, Drenth J, McIntyre CL (2014). The heat shock factor family from Triticum aestivum in response to heat and other major abiotic stresses and their role in regulation of heat shock protein genes. J Exp Bot.

[30] Xue GP, Drenth J, McIntyre CL (2015). TaHsfA6f is a transcriptional activator that regulates a suite of heat stress protection genes in wheat (Triticum aestivum L.) including previously unknown Hsf targets. J Exp Bot.

[31] Agarwal P, Khurana P (2019). Functional characterization of HSFs from wheat in response to heat and other abiotic stress conditions. Funct Integr Genomics.

[32] Hu XJ, Chen D, Lynne Mclntyre C, Fernanda Dreccer M, Zhang ZB, Drenth J, Kalaipandian S, Chang H, Xue GP (2018). Heat shock factor C2a serves as a proactive mechanism for heat protection in developing grains in wheat via an ABA-mediated regulatory pathway. Plant Cell Environ.

[33] Zhao LN, Liu ZH, Duan SN, Zhang YY, Li GL, Guo XL (2018). Cloning and Characterization of heat shock transcription factor gene TaHsfB2d and its regulating role in thermotolerance. Acta Agron Sin.

[34] Zhang YJ, Zhang YY, Zhang HN, Qin N, Li GL, Guo XL (2018). Characterization and regulatory roles in thermotolerance of wheat heat shock transcription factor gene TaHsfA2e. Acta Agron Sin.

[35] Core writing team. In: Arneth AF, Denton F, editors. Special report: climate change and land. Farming and context: IPCC: Climate Change and land, Cambridge University Press; 2019.

[36] Asseng S, Ewart F, Martre P, Rotter RP, Lobell DB, Cammarano D, Kimball BA, Ottman MJ, Wall GW, White JW (2014). Rising temperatures reduce global wheat production. Nat Clim Chang.

[37] Pauli H, Gottfried M, Dullinger S (2012). Recent plant diversity changes on Europe’s mountain summits. Science..

[38] Lobell DB, Schlenker W, Costa-Roberts J (2011). Climate trends and global crop production since 1980. Science..

[39] Liu J, Feng L, Li J, He Z (2015). Genetic and epigenetic control of plant heat responses. Front Plant Sci.

[40] Larkindale J, Hall JD, Knight MR, Vierling E (2005). Heat stress phenotypes of Arabidopsis mutants implicate multiple signaling pathways in the acquisition of thermotolerance. Plant Physiol.

[41] Huang YC, Niu CY, Yang CR, Jinn TL (2017). The heat stress factor HsfA6b connects ABA signaling and ABA-mediated heat responses. Plant Physiol.

[42] Ikeda M, Mitsuda N, Ohme-Takagi M (2011). Arabidopsis HsfB1 and HsfB2b act as repressors for the expression of heat-inducible Hsfs but positively regulate the acquired thermotolerance. Plant Physiol.

[43] Ma H, Wang CT, Yang B, Cheng HY, Wang Z, Mijiti A, Ren C, Qu GH, Zhang H, Ma L (2016). CarHSFB2, a Class B Heat shock transcription factor, is involved in different developmental processes and various stress responses in chickpea (Cicer arietinum L.). Plant Mol Biol Report.

[44] Snyman M, Cronje MJ (2008). Modulation of heat shock factors accompanies salicylic acid-mediated potentiation of Hsp70 in tomato seedlings. J Exp Bot.

[45] Agashe VR, Hartl FU (2000). Roles of molecular chaperones in cytoplasmic protein folding. Semin Cell Dev Biol.

[46] Downs CA, Heckathorn SA (1998). The mitochondrial small heat-shock protein protects NADH:ubiquinone oxidoreductase of the electron transport chain during heat stress in plants. FEBS Lett.

[47] Heckathorn SA, Downs CA, Sharkey TD, Coleman JS (1998). The small, methionine-rich chloroplast heat-shock protein protects photosystem II electron transport during heat stress. Plant Physiol.

[48] Charng YY, Liu HC, Liu NY, Hsu FC, Ko SS (2006). Arabidopsis Hsa32, a novel heat shock protein, is essential for acquired thermotolerance during long recovery after acclimation. Plant Physiol.

[49] Lin MY, Chai KH, Ko SS, Kuang LY, Lur HS, Charng YY (2014). A positive feedback loop between heat shock protein101 and heat stress-associated 32-KD protein modulates long-term acquired thermotolerance illustrating diverse heat stress responses in rice varieties. Plant Physiol.

[50] Zhang YY, Zhao H, Zhang YJ, Duan SN, Li GL (2019). Biological characteristics and thermotolerance-regulating roles of wheat (Triticum aestivum) heat shock transcription factor gene TaHsfA2f. J Agri Biotech.

[51] Chen, C., Xia, R., Chen, H. & He, Y. TBtools, a toolkit for biologists integrating various HTS-data handling tools with a user-friendly interface. BioRxiv 289660. 2018;10.1101/289660.

[52] Clough SJ, Bent AF (1998). Floral dip: a simplified method for Agrobacterium-mediated transformation of Arabidopsis thaliana. Plant J.

[53] Li HC, Zhang HN, Li GL, Liu ZH, Zhang YM, Zhang HM, Guo XL (2015). Expression of maize heat shock transcription factor gene ZmHsf06 enhances the thermotolerance and drought-stress tolerance of transgenic Arabidopsis. Funct Plant Biol.

