# Supplementary figures and images for: Heat-response patterns of the heat shock transcription factor family in advanced development stages of wheat (Triticum aestivum L.) and thermotolerance-regulation by TaHsfA2–10
Source: BMC Plant Biol. 2020 Aug 3;20:364. doi: 10.1186/s12870-020-02555-5 (PMC7397617; doi:10.1186/s12870-020-02555-5)

Fig 6. A

TaHsfA2-10


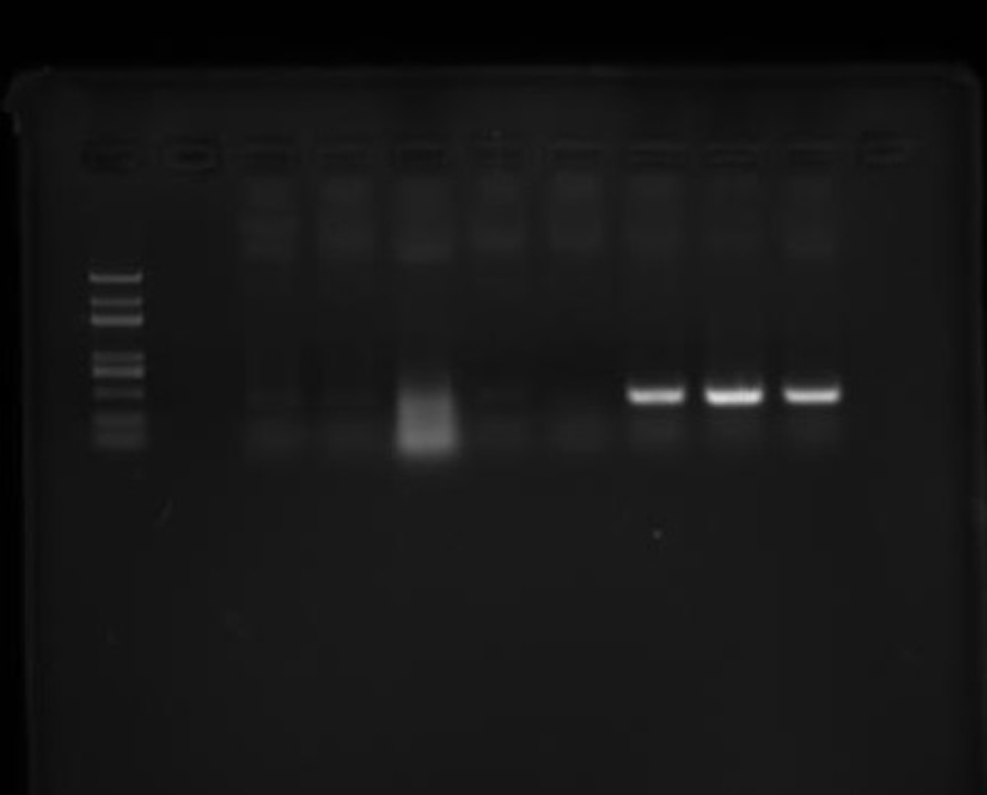


Atactin8


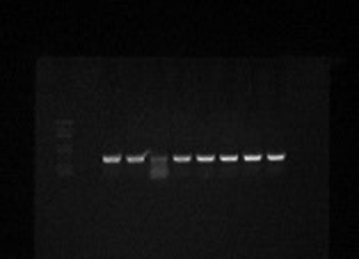


Fig 7. A

TaHsfA2-10


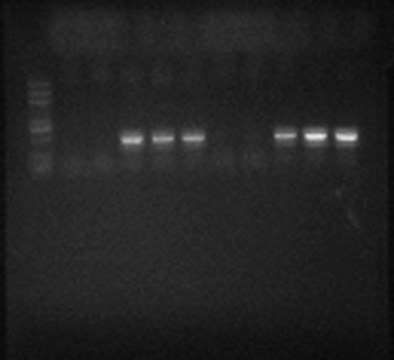


Atactin8


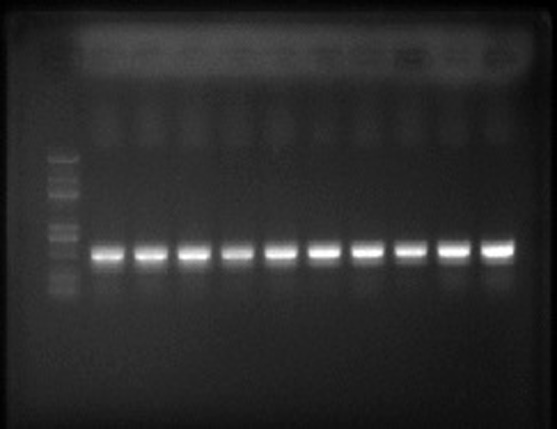

Supplement: Supplementary file 3 — Additional file 3. Original, unprocessed versions of the blots in Figs. 6 and 7. [file 12870_2020_2555_MOESM3_ESM.docx]
