# Supplementary material for: Heat-response patterns of the heat shock transcription factor family in advanced development stages of wheat (Triticum aestivum L.) and thermotolerance-regulation by TaHsfA2–10
Source: BMC Plant Biol. 2020 Aug 3;20:364. doi: 10.1186/s12870-020-02555-5 (PMC7397617; doi:10.1186/s12870-020-02555-5)
Supplement: Supplementary file 4 — Additional file 4 The primers for Arabidopsis Hsp genes related to thermotolerance in qRT-PCR. [file 12870_2020_2555_MOESM4_ESM.docx]

**Additional file 3.** The primers for *Arabidopsis* Hsp genes related to thermotolerance in qRT-PCR

| Gene | Primer (5’-3’) | Annealing temperature | Product size (bp) | Purpose |
| --- | --- | --- | --- | --- |
| *AtHsa32* | Forward: GCGAAGTTGGTTGAGTGGTT | 60°C | 138 | Gene expression |
|  | Reverse: GGAGGAACTGAGAACAGATTGG |  |  |  |
| *AtERDJ3A* | Forward: CTCCTGTTTGTATCATTGGTGC | 60°C | 128 | Gene expression |
|  | Reverse: TGTGTCCTGAGAACCTGTGG |  |  |  |
| *AtHsp70T* | Forward: TGATTGAGGTGAGGATGCC | 60°C | 231 | Gene expression |
|  | Reverse: CCACTTCAACGACAAACCC |  |  |  |
| *AtHsp90.1* | Forward: CCCTCTCTTCTTCATAAATCAACA | 60°C | 125 | Gene expression |
|  | Reverse: CCATCGCAACGAACTTTG |  |  |  |
| *AtHsp101* | Forward: TGTCTTCAACACTCTGCTCCA | 60°C | 157 | Gene expression |
|  | Reverse: CACTTCCATTGTTACTTTCCCAG |  |  |  |
| *AtActin8* | Forward: GCCAGATCTTCATCGTCGTG | 60°C | 161 | Internal reference |
|  | Reverse: TCTCCAGCGAATCCAGCCTT |  |  |  |
