# Supplementary material for: Heat-response patterns of the heat shock transcription factor family in advanced development stages of wheat (Triticum aestivum L.) and thermotolerance-regulation by TaHsfA2–10
Source: BMC Plant Biol. 2020 Aug 3;20:364. doi: 10.1186/s12870-020-02555-5 (PMC7397617; doi:10.1186/s12870-020-02555-5)
Supplement: Supplementary file 5 — Additional file 5 The primers of the promoters of AtHsps used in yeast one hybrid. [file 12870_2020_2555_MOESM5_ESM.docx]

**Additional file 4.** The primers of the promoters of *AtHsps* used in yeast one hybrid

| Promoter | Primer (5’-3’) | Annealing temperature | Product size (bp) | Purpose |
| --- | --- | --- | --- | --- |
| *AtHsa32* | Forward: GACTCACTATAGGGCGAATTCAATAACTTGTTCTTGT | 62°C | 684 | Binding activity |
|  | Reverse: ATGCCAGGAATTACTAGTTTTCTCTGTTCACTTC |  |  |  |
| *AtERDJ3A* | Forward: GACTCACTATAGGGCGAATTCAATTTCTGCCTC | 62°C | 397 | Binding activity |
|  | Reverse: ATGCCAGGAATTACTAGTAATCTATGTGTC |  |  |  |
| *AtHsp70T* | Forward: GACTCACTATAGGGCGAATTCGAAACTGAAAAAG | 62°C | 386 | Binding activity |
|  | Reverse: ATGCCAGGAATTACTAGTGAGCGTCAAAGC |  |  |  |
| *AtHsp90.1* | Forward: GACTCACTATAGGGCGAATTCAGAAGACAAATGAGA | 62°C | 454 | Binding activity |
|  | Reverse: ATGCCAGGAATTACTAGTCTGAACATCCGCCATC |  |  |  |
| *AtHsp101* | Forward: GACTCACTATAGGGCGAATTCCAATTAGCCAAGTTC | 62°C | 451 | Binding activity |
|  | Reverse: ATGCCAGGAATTACTAGTGATTATAGCGGTAATG |  |  |  |

Underlined letters were restriction enzyme sites *Eco*RI or *Spe*I.
