# Supplementary material for: Heat-response patterns of the heat shock transcription factor family in advanced development stages of wheat (Triticum aestivum L.) and thermotolerance-regulation by TaHsfA2–10
Source: BMC Plant Biol. 2020 Aug 3;20:364. doi: 10.1186/s12870-020-02555-5 (PMC7397617; doi:10.1186/s12870-020-02555-5)
Supplement: Supplementary file 6 — Additional file 6. The vector maps used in this paper. [file 12870_2020_2555_MOESM6_ESM.pdf]

A

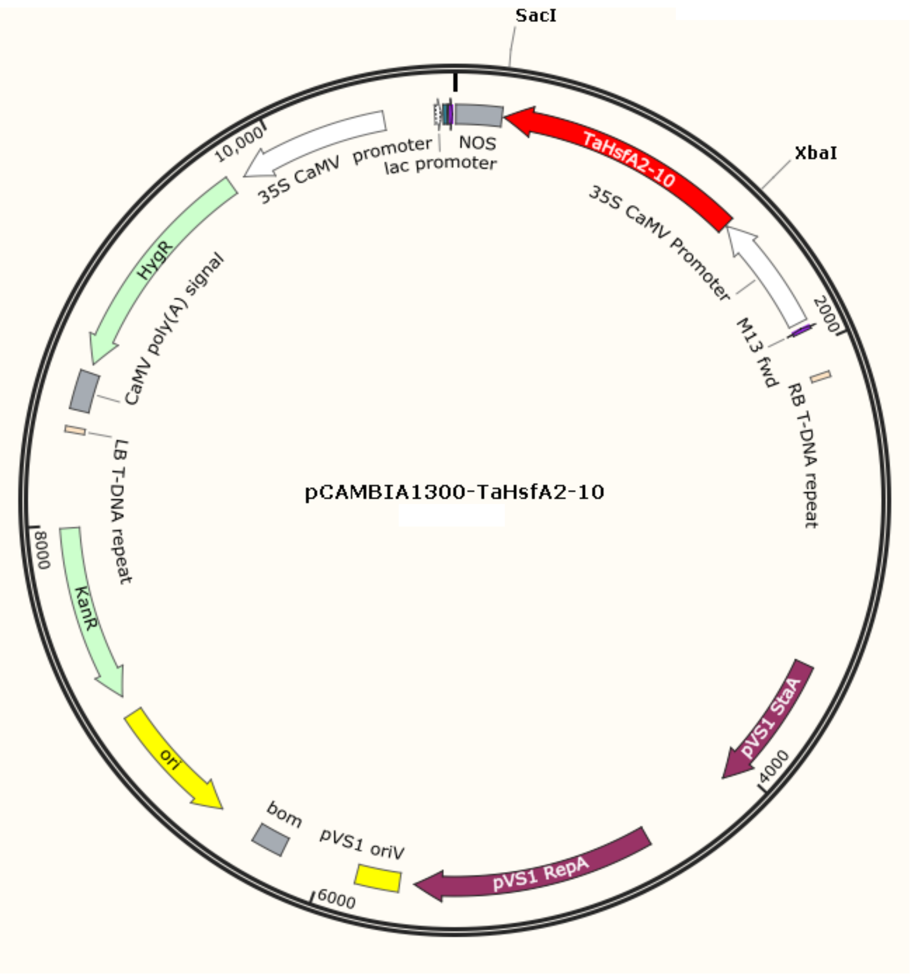

The vector map of pCAMBIA1300-TaHsfA2-10

B

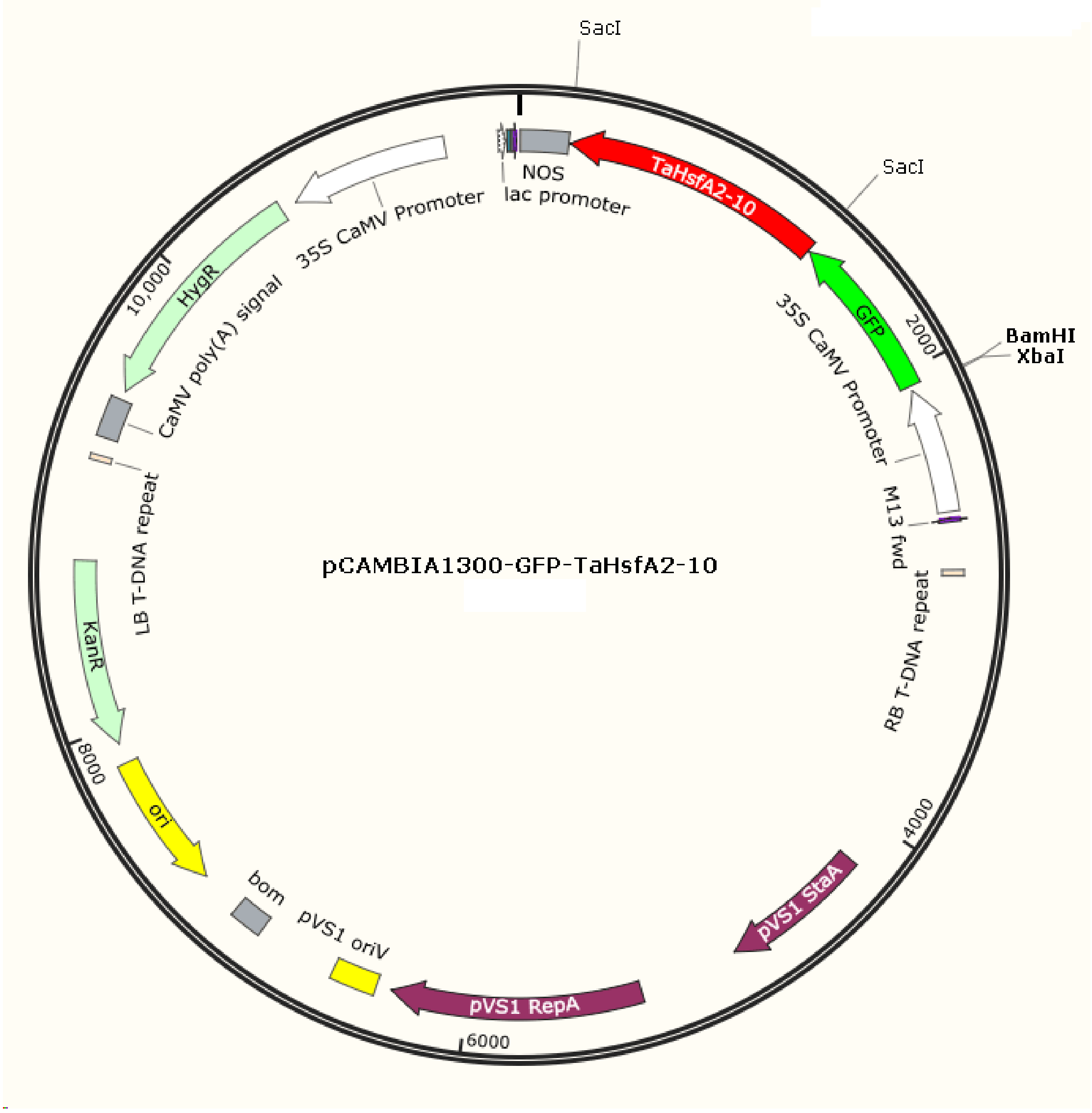

The vector map of pCAMBIA1300-GFP-TaHsfA2-10

C

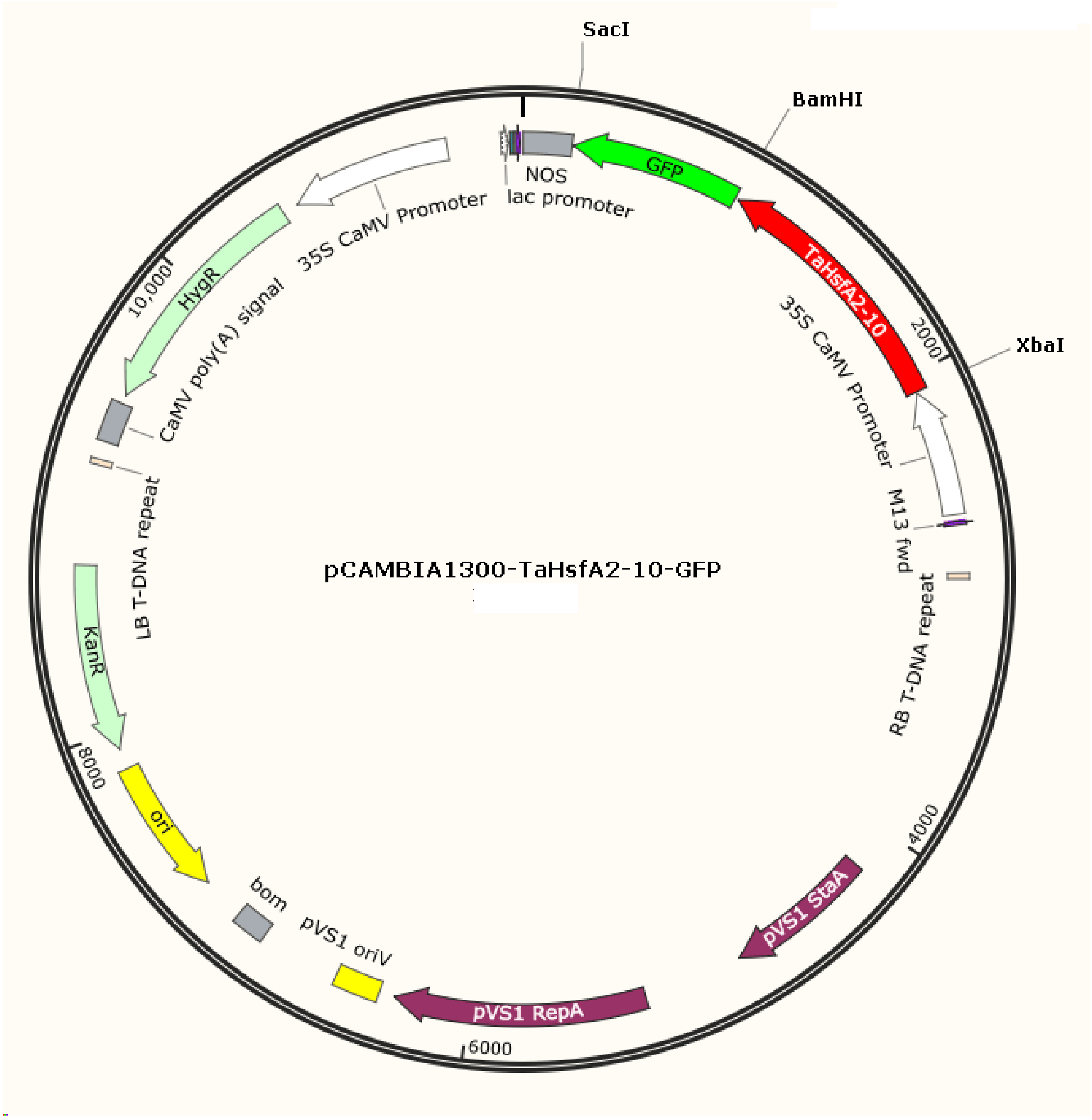

The vector map of pCAMBIA1300-TaHsfA2-10-GFP

D

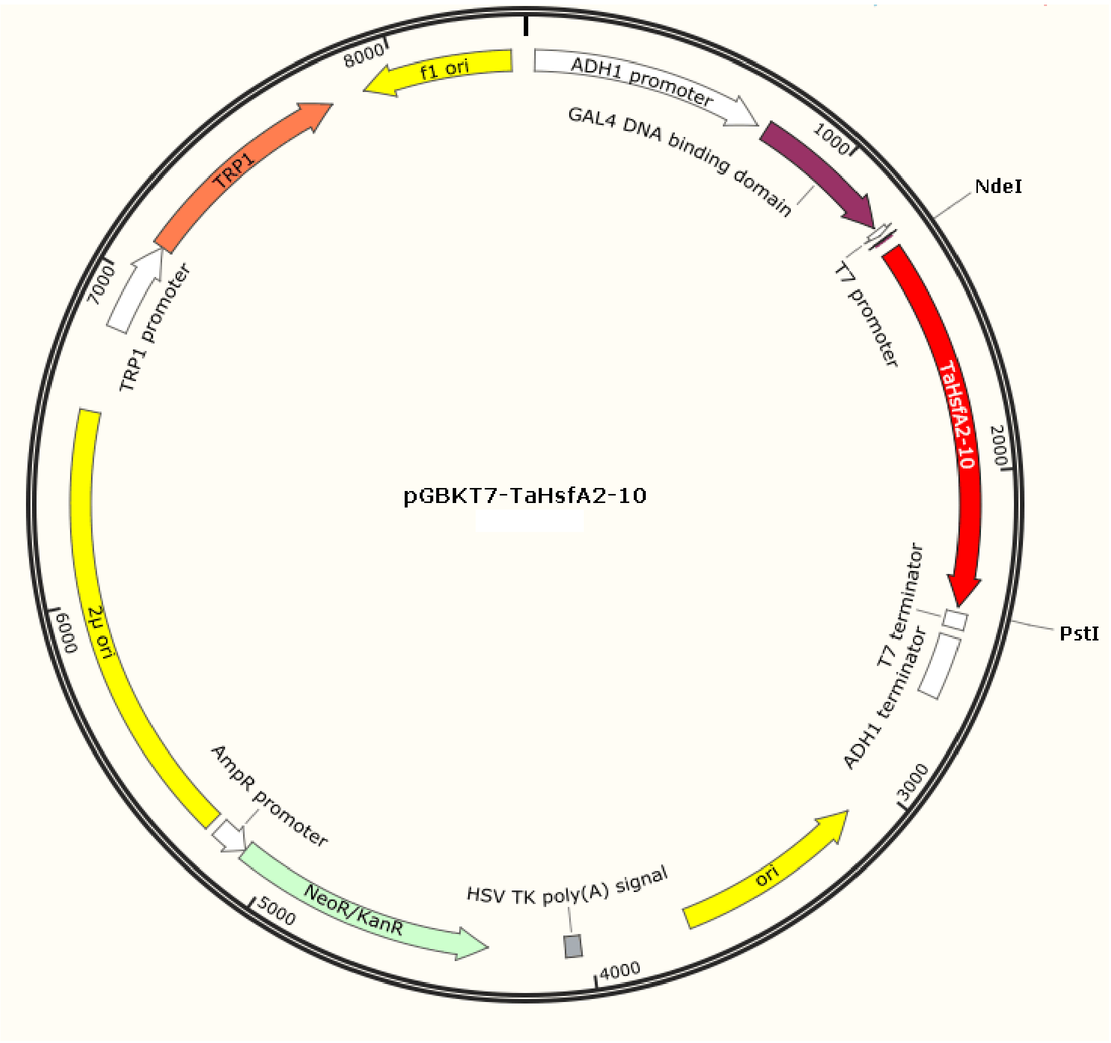

The vector map of pGBKT7-TaHsfA2-10

E

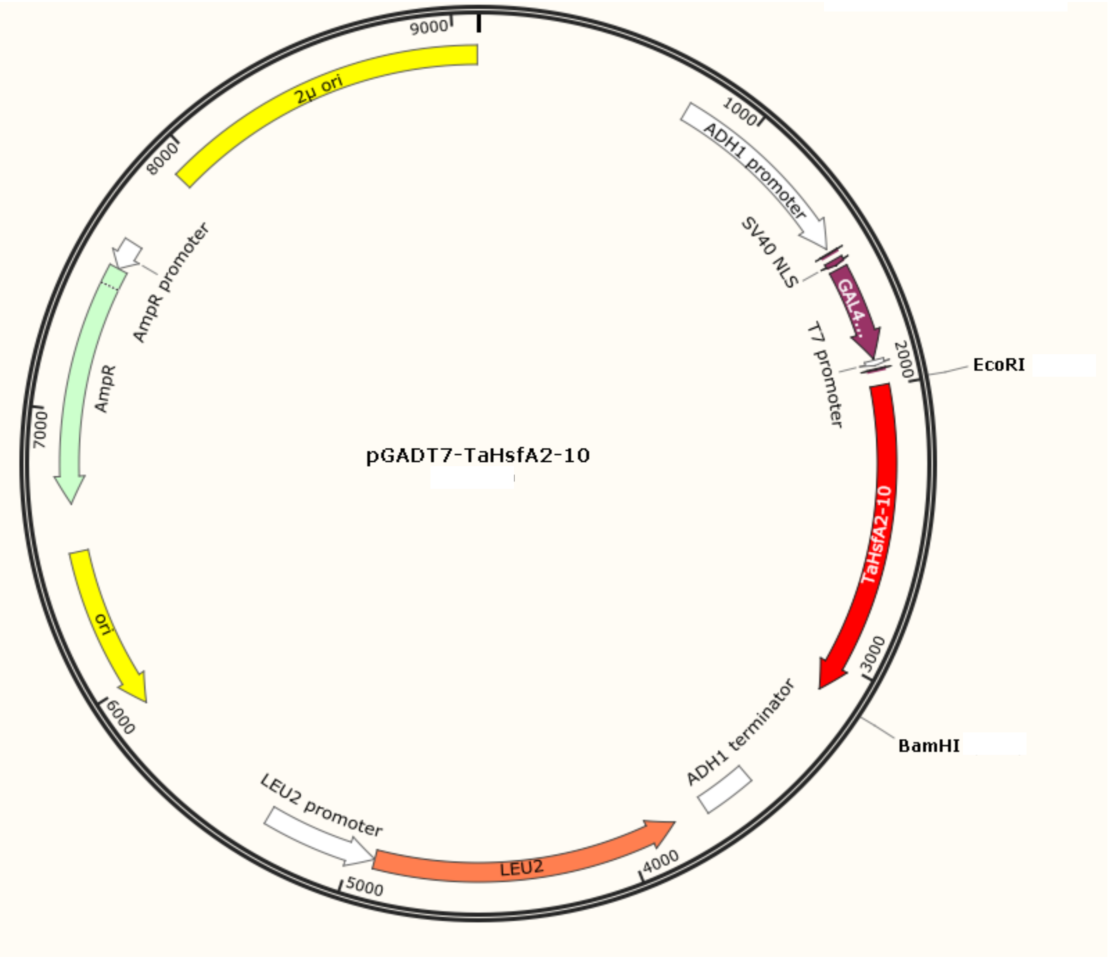

The vector map of pGADT7-TaHsfA2-10

F

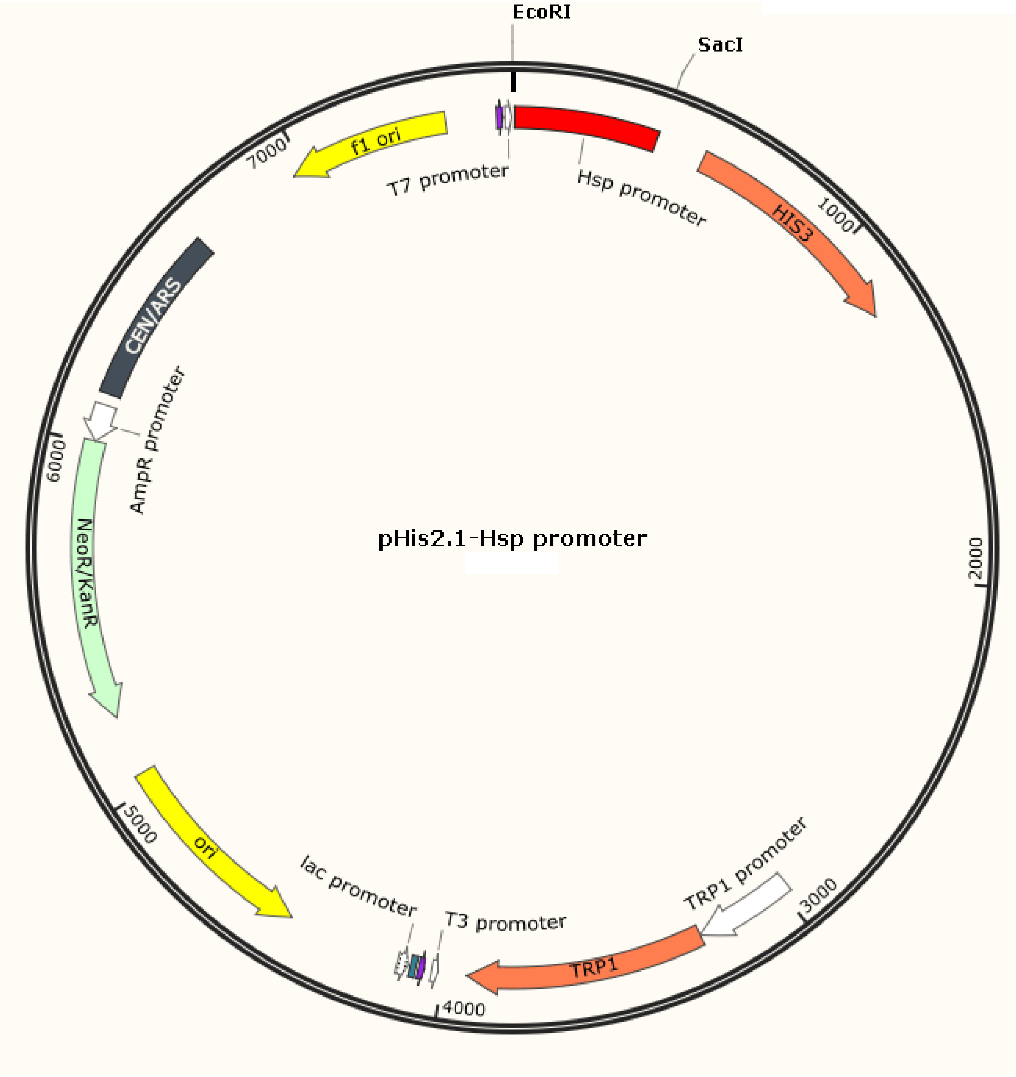

The vector map of pHIS2.1-Hsp promoter
